# Clinical outcomes of different sclerosing agents for hemorrhoids: a network meta-analysis

**DOI:** 10.3389/fmed.2026.1915729

**Published:** 2026-10-07

**Authors:** Bin Chen, Zhenguo Zhang, Xinru Xiong, Chilei Shen

**Affiliations:** Department of Proctology, Ningbo Hospital of Integrated Traditional Chinese and Western Medicine, Ningbo, China

**Keywords:** cure rate, hemorrhoids, network meta-analysis, polidocanol foam, sclerotherapy

## Abstract

**Background:**

Injection sclerotherapy is widely used for grade I–III internal hemorrhoids, but evidence comparing multiple sclerosing agents head-to-head is limited.

**Methods:**

Pubmed, Web of Science, Embase, Cochrane Library, CNKI, Wanfang Data, and grey literature sources were searched for randomized controlled trials (RCTs) reporting cure rate as the primary outcome. A Bayesian network meta-analysis was performed using Markov chain Monte Carlo simulations. Odds ratios (ORs) with 95% confidence intervals (CIs) were calculated, and treatments were ranked using surface under the cumulative ranking curve (SUCRA) values. Outcomes assessed included cure rate, recurrence rate, patient satisfaction, and operative complications.

**Results:**

Sixty-six RCTs were included. Polidocanol foam demonstrated significantly higher cure rates compared with polidocanol liquid (OR = 3.1, 95% CI: 2.3–4.2) and ranked first among all treatments for cure rate and patient satisfaction. It also showed the lowest risk of overall complications (OR = 0.53 vs. polidocanol liquid, 95% CI: 0.22–1.3), with significantly reduced risks of bleeding (OR = 0.4, 95% CI: 0.21–0.9) and rectal stricture (OR < 0.001, 95% CI: <0.001–0.73). XiaoZhiLing, phenol, and 50% dextrose were significantly inferior to polidocanol liquid in cure rate. Subgroup analyses in internal hemorrhoids, grade I/II, and grade I/II/III hemorrhoids consistently supported the superiority of polidocanol foam.

**Conclusion:**

Polidocanol foam showed best efficacy among all sclerosing agents for treating hemorrhoids, ranking first in cure rate and patient satisfaction with lowest complication risks, particularly for bleeding and rectal stricture. These findings support polidocanol foam as the preferred sclerosing agent in clinical practice. However, as most included trials had follow-up durations of less than 1 year, the long-term durability of these benefits and the risk of late recurrence remain to be established.

**Systematic Review Registration:**

https://www.crd.york.ac.uk/PROSPERO/view/CRD420261379386, PROSPERO CRD420261379386.

## Introduction

Hemorrhoids are among the most common anorectal disorders worldwide, with a reported prevalence ranging from 16% to 70% in the general adult population (1–3). The condition significantly impairs patients' quality of life due to distressing symptoms including rectal bleeding, prolapse, pain, pruritus, and discomfort during defecation (4, 5). According to current clinical guidelines, a step-up approach is recommended: asymptomatic hemorrhoids require no active intervention, whereas symptomatic patients should initially receive conservative treatments such as dietary modification and lifestyle changes. When conservative measures fail, office-based or surgical interventions are indicated. Commonly used modalities include rubber band ligation (RBL), injection sclerotherapy, procedure for prolapse and hemorrhoids (PPH), laser therapy, and conventional hemorrhoidectomy (6). Each approach carries its own balance of efficacy and complications—for example, hemorrhoidectomy offers low recurrence but significant postoperative pain, while less invasive options may have higher recurrence rates. Among these, injection sclerotherapy has emerged as a particularly attractive option for grade I–III internal hemorrhoids due to its simplicity, outpatient feasibility, minimal post-procedural discomfort, low complication rates, and favorable risk-benefit profile (7, 8).

Despite the widespread use of injection sclerotherapy for hemorrhoids, a variety of sclerosing agents have been used in clinical practice, including polidocanol (available in both liquid and foam formulations), phenol, 50% dextrose, and traditional Chinese medicine-based agents such as XiaoZhiLing, ShaoBei, and FanTengZhi (9–13). These agents differ substantially in their mechanisms of action, optimal concentrations, injection techniques, and clinical outcomes, leading to considerable heterogeneity in reported efficacy and safety profiles. For instance, foam formulations may provide better mucosal contact and lower required doses compared with liquid forms, while different chemical compositions exhibit varying degrees of local inflammatory response and fibrotic effect. Importantly, existing randomized controlled trials (RCTs) have primarily focused on head-to-head comparisons between two specific sclerosing agents or between a single sclerosing agent and other modalities such as RBL or conventional hemorrhoidectomy (14–16). Consequently, a systematic comparison that simultaneously evaluates multiple sclerosing agents within a unified analytical framework remains lacking. This evidence gap poses a practical challenge for clinicians, who must select among various sclerosing agents without sufficient high-grade evidence to guide their decision-making.

Although several systematic reviews have attempted to synthesize evidence on sclerotherapy for hemorrhoids, most have been limited to conventional pairwise meta-analyses, which can only perform direct comparisons between two interventions (10, 17, 18). This approach cannot address clinically relevant questions involving interventions that have never been directly compared in head-to-head trials. Even in systematic reviews that include multiple sclerosing agents, the analyses are typically conducted as separate pairwise meta-analyses without integrating indirect evidence or providing a hierarchical ranking of treatment efficacy. Network meta-analysis (NMA) can overcome these limitations by combining both direct and indirect evidence within a unified framework, enabling simultaneous comparisons across multiple interventions and generating rankings of treatment effects. Furthermore, existing studies often overlook the inclusion of common non-sclerotherapy comparators such as conventional hemorrhoidectomy, PPH, and RBL, which serve as crucial bridges for indirect comparisons among sclerosing agents. To date, no network meta-analysis has been specifically designed to systematically compare the clinical outcomes of different sclerosing agents for hemorrhoids. Therefore, the present study aims to address this gap by conducting a comprehensive NMA evaluating various sclerosing agents, such as polidocanol liquid, polidocanol foam, phenol, 50% dextrose, XiaoZhiLing, ShaoBei, and FanTengZhi, with conventional hemorrhoidectomy, coagulation, PPH, and RBL serving as common comparators to enhance the validity and reliability of indirect comparisons.

## Methods

### Study design and search strategy

This network meta-analysis was conducted in accordance with the Preferred Reporting Items for Systematic Reviews and Meta-Analyses (PRISMA) statement (19). The protocol of this study was registered on PROSPERO (registration number: 1379386). A comprehensive literature search was performed to identify eligible randomized controlled trials evaluating the clinical efficacy of different sclerosing agents as well as conventional surgical treatments for hemorrhoids. Conventional hemorrhoidectomy, procedure for prolapse and hemorrhoids (PPH), and rubber band ligation (RBL) were included as comparators to enhance the reliability of indirect comparisons, as these three modalities represent the most commonly used treatments in randomized controlled trials against sclerotherapy for hemorrhoids. The following electronic databases were searched from inception to April 2026: PubMed (Medline), Web of Science, Embase, Cochrane Library, China National Knowledge Infrastructure (CNKI), and Wanfang Data. In addition, grey literature was identified through a search of Google Scholar, ClinicalTrials.gov, and the reference lists of relevant systematic reviews and original studies.

The search strategy combined MeSH terms and free-text words related to three domains: (1) hemorrhoids (e.g., “hemorrhoids,” “haemorrhoids,” “piles”); (2) interventions of interest, including sclerotherapy (e.g., “sclerotherapy,” “sclerosing agents,” “lauromacrogol,” “polidocanol,” “XiaoZhiLing”), conventional hemorrhoidectomy, PPH, and RBL; and (3) study types (e.g., “randomized controlled trial,” “clinical trial”). For Chinese databases, both Chinese and English keywords were used to ensure comprehensive retrieval. No language or publication date restrictions were applied.

### Eligibility criteria and study selection

Studies were considered eligible for inclusion in this network meta-analysis if they met all of the following criteria based on the PICOS framework:

Population (P): Adult patients with a confirmed diagnosis of hemorrhoids, regardless of grade (I–IV), sex, ethnicity, or disease duration.
Interventions (I) and Comparators (C): The intervention of interest was any sclerosing agent, including but not limited to lauromacrogol foam, polidocanol injection, and XiaoZhiLing injection. Eligible comparators included: Other sclerosing agents (head-to-head comparisons), conventional hemorrhoidectomy, PPH, or RBL.
Outcomes (O): Primary outcome is cure rate. Studies that did not report cure rate as an outcome were excluded, even if they reported other outcomes. Secondary outcomes: Recurrence rate, patient satisfaction rate, and postoperative complications.
Study design (S): Only RCTs were included. Non-randomized studies, case series, case reports, reviews, meta-analyses, animal experiments, and basic cell studies were excluded.
Two reviewers independently screened the titles and abstracts of all retrieved records, followed by a full-text assessment against predefined eligibility criteria. Disagreements were resolved by discussion or consultation with a third reviewer.

### Data extraction

Two reviewers independently extracted data from all included studies using a standardized form. The following information was collected: first author, year of publication, hemorrhoid type, hemorrhoid grade, sample size, details of interventions, and outcome data. The primary outcome was cure rate. Given anticipated heterogeneity in outcome definitions across studies, the cure rate was defined based on any of the following criteria: Complete resolution of hemorrhoidal symptoms (e.g., bleeding, prolapse, pain, pruritus) and/or complete involution of hemorrhoidal cushions as assessed by proctoscopy; a validated scale, such as the Soddo Hemorrhoid Severity Score (SHSS) = 0 and Hemorrhoidal Disease and Bleeding Grade (HDBG) ≤1; or any equivalent definition indicating the absence of both symptoms and objective signs of hemorrhoids. Secondary outcomes included recurrence rate, patient satisfaction, and postoperative complications. All outcomes were binary variables. For each outcome, we extracted the number of events and the total number of patients in each treatment arm. Disagreements were resolved by discussion or a third reviewer.

### Risk of bias assessment

Two independent reviewers assessed the methodological quality of included studies using the Cochrane RoB 2.0 tool (20). The tool evaluates five domains: randomization process, deviations from intended interventions, missing outcome data, measurement of the outcome, and selection of reported results. Each domain was judged as “low risk,” “some concerns,” or “high risk.” Disagreements were resolved by discussion or by consulting a third reviewer.

### Statistical analysis

All analyses were performed using R software (version 4.3.0) with the gemtc and rjags packages. A Bayesian network meta-analysis was conducted using Markov chain Monte Carlo (MCMC) simulations. Four chains were run with 50,000 adaptation iterations, 50,000 burn-in iterations, and 100,000 post-burn-in iterations with a thinning interval of 10. Convergence was assessed using the Brooks-Gelman-Rubin diagnostic (potential scale reduction factor <1.05). A consistency model was fitted assuming consistency between direct and indirect evidence. Odds ratio (OR) was calculated to present the effect size of comparisons. Global inconsistency was evaluated using the design-by-treatment interaction model, and local inconsistency was assessed using the node-splitting method. Heterogeneity was explored via the between-study variance (*τ*^2^). Treatment rankings were determined using surface under the cumulative ranking curve (SUCRA) values, with results presented as rankograms and a league table. Subgroup analyses on cure rate were performed in three populations: patients with internal hemorrhoids, those with grade I/II hemorrhoids, and those with grade I/III hemorrhoids.

## Results

### Study characteristics and risk of bias assessment

A total of 3,430 records were identified through database searches and grey literature sources. After removing 1,952 duplicates, 1,478 records were screened. Following title and abstract screening, 1,236 reports were sought for retrieval, of which 640 underwent full-text assessment. Ultimately, 66 studies met the eligibility criteria and were included in this network meta-analysis. The study selection process is summarized in Figure 1.

**Figure 1:**
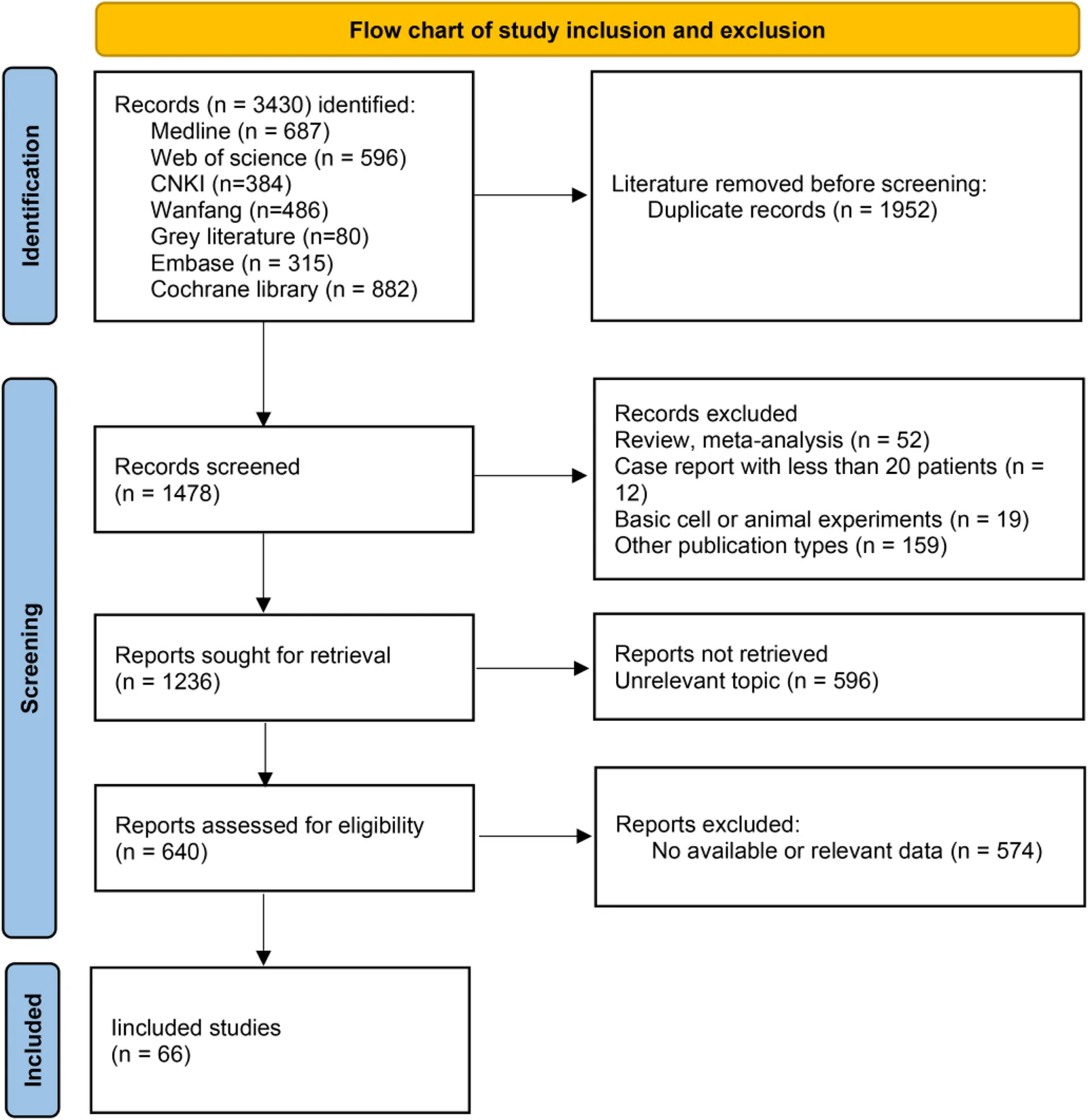
PRISMA flow diagram of study selection. A total of 3,430 records were identified from eight databases and grey literature sources. After removing 1,952 duplicates, 1,478 records were screened. Following title and abstract screening, 1,236 reports were sought for retrieval, of which 640 underwent full-text assessment. Ultimately, 66 randomized controlled trials met the eligibility criteria and were included in the network meta-analysis.

The characteristics of all included studies are presented in Supplementary Table S1, and the full reference list is provided in the Supplementary Materials. Among the 66 included studies, all were RCTs. The sample sizes ranged from 23 to 385 participants across treatment arms. Regarding hemorrhoid types, most studies focused on internal hemorrhoids (*n* = 56), followed by mixed hemorrhoids (*n* = 4), and unspecified or unrestricted types (*n* = 6). The grade of hemorrhoids varied: 7 studies included grade I/II, 19 included grade I/II/III, 13 included grade II/III, 5 included grade II alone, 3 included grade I alone, and the remaining studies reported all grades or did not specify. Interventions included various sclerosing agents, such as polidocanol (liquid or foam), phenol, 50% dextrose, as well as traditional Chinese medicine sclerosing agents (XiaoZhiLing, ShaoBei, and FanTengZhi, with detailed compositions and active ingredients provided in Supplementary Materials). Comparators included coagulation, traditional hemorrhoidectomy, PPH, and RBL.

Risk of bias was assessed using the Cochrane RoB 2.0 tool for each included study (Supplementary Table S1). Across the 66 RCTs, the majority demonstrated low risk of bias in most domains. Bias arising from the randomization process was the main source of concern, primarily because many studies stated that they were randomized but did not describe the specific randomization method. Overall, the included studies were considered to have acceptable methodological quality for the network meta-analysis.

### Cure rate

The network connections among different interventions for cure rate are illustrated in Figure 2. Polidocanol liquid was the most frequently studied agent (*n* = 40), followed by RBL (*n* = 22), XiaoZhiLing (*n* = 21), and polidocanol foam (*n* = 19). ShaoBei was only directly compared with XiaoZhiLing, while all other treatments had head-to-head comparisons with at least two other interventions.

**Figure 2:**
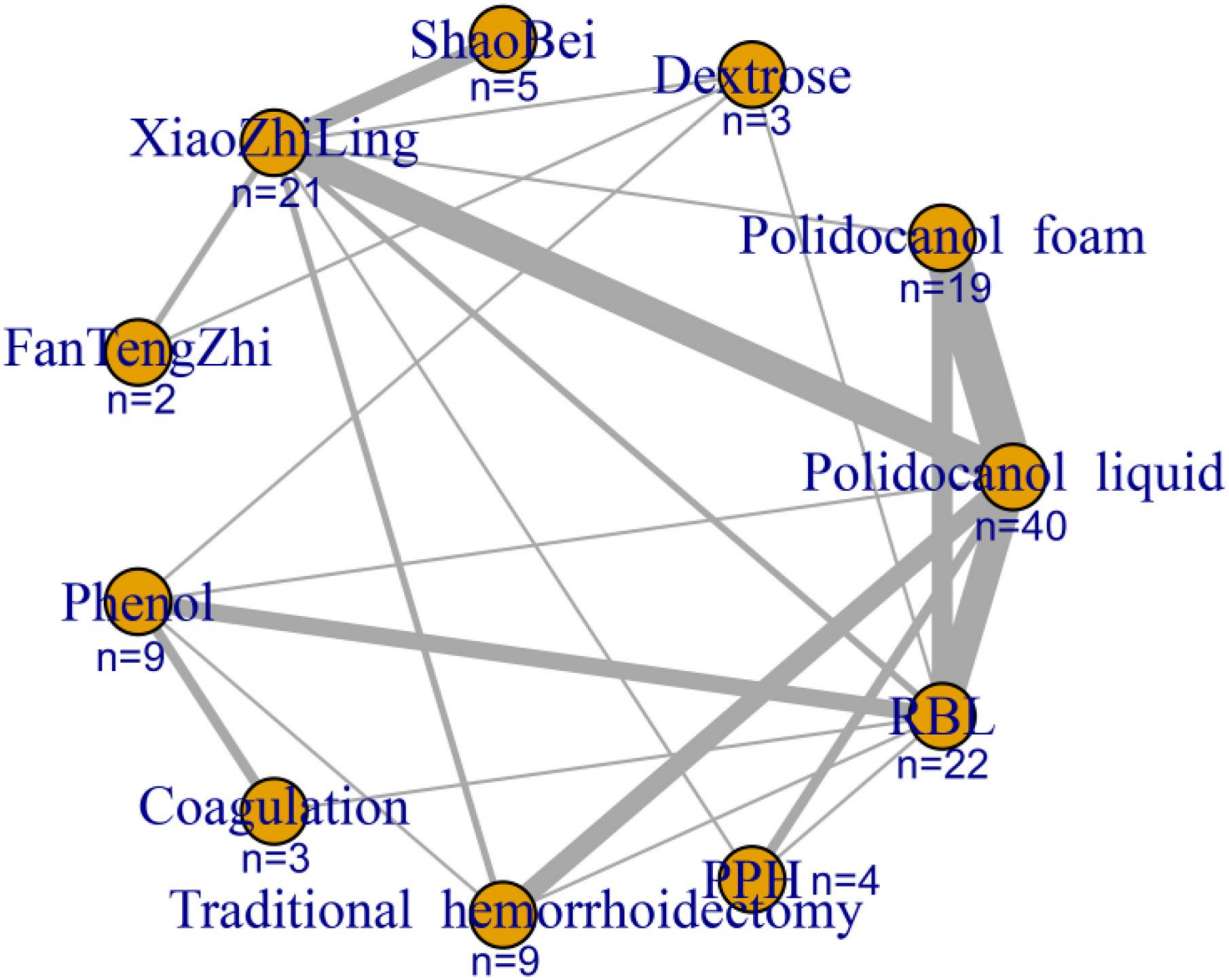
Network plot of treatment comparisons for cure rate. Each node represents an intervention, and the number beside each node (*n* = xxx) indicates the number of randomized controlled trials evaluating that intervention. Edges connect interventions that have been directly compared in head-to-head trials, with edge thickness corresponding to the number of direct comparisons. RBL, rubber band ligation; PPH, procedure for prolapse and hemorrhoids.

Figure 3 presents the forest plot using polidocanol liquid as the reference, as it is a commonly used sclerosing agent in clinical practice. Polidocanol foam demonstrated a significant advantage in cure rate compared with polidocanol liquid (OR = 3.1, 95% CI: 2.3–4.2). Several other treatments showed similar cure rates to polidocanol liquid without significant differences, including ShaoBei (OR = 1.4, 95% CI: 0.78–2.6) and FanTengZhi (OR = 0.94, 95% CI: 0.31–3.1). In contrast, dextrose (OR = 0.35, 95% CI: 0.12–0.99), XiaoZhiLing (OR = 0.49, 95% CI: 0.35–0.7), and phenol (OR = 0.42, 95% CI: 0.24–0.72) were significantly inferior to polidocanol liquid.

**Figure 3:**
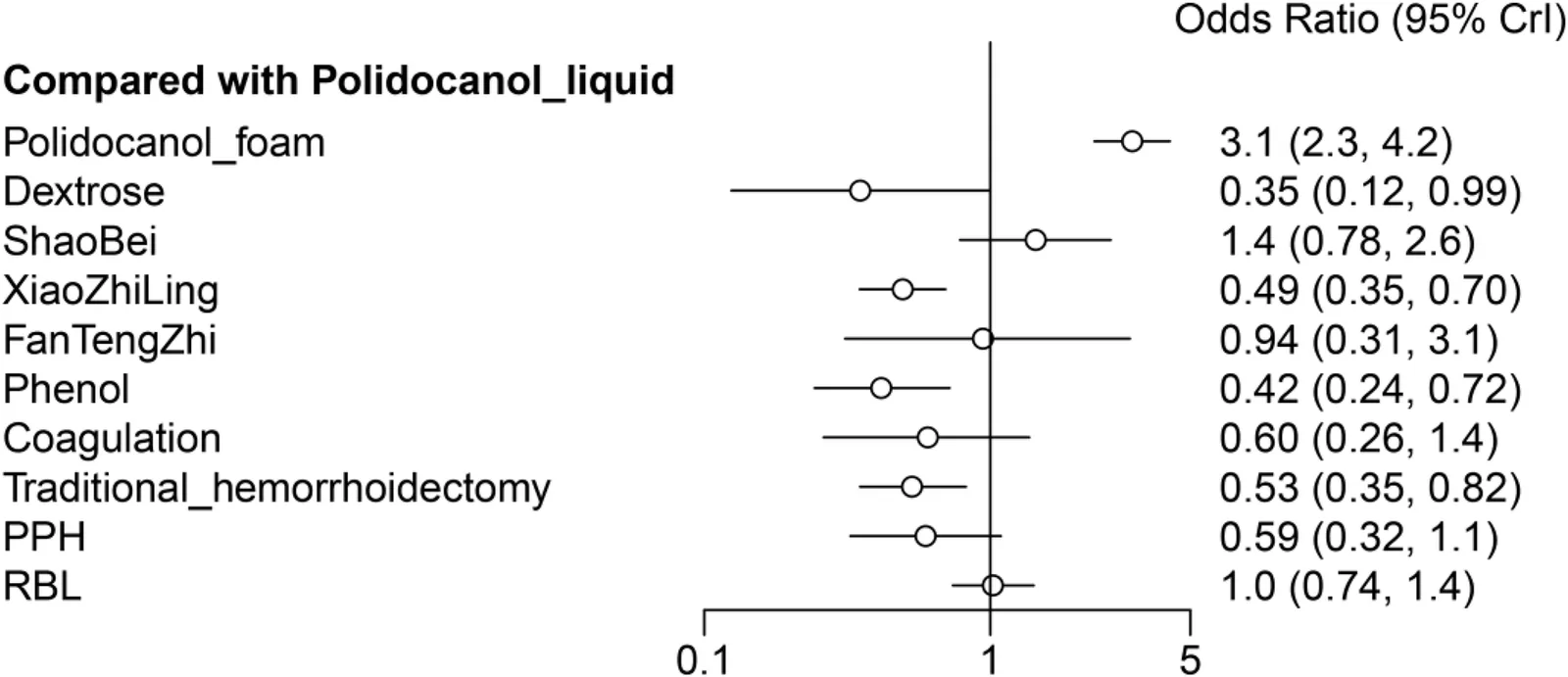
Forest plot of cure rate with polidocanol liquid as reference. Odds ratios (ORs) and 95% CIs are shown for each intervention compared with polidocanol liquid. An OR greater than 1 favors the experimental intervention, whereas an OR less than 1 favors polidocanol liquid. PPH, procedure for prolapse and hemorrhoids; RBL, rubber band ligation.

Convergence diagnostics of the network meta-analysis are shown in Supplementary Figure S1. The potential scale reduction factors (PSRF) for all comparisons ranged from 1.00 to 1.01, indicating that the model converged adequately and the estimates were reliable.

The ranking plot further supported the above findings (Figure 4). Polidocanol foam had the highest probability of being the best treatment (rank 1), followed by ShaoBei (rank 2) and polidocanol liquid (rank 3). Detailed ORs for all pairwise comparisons are presented in the league table (Table 1).

**Figure 4:**
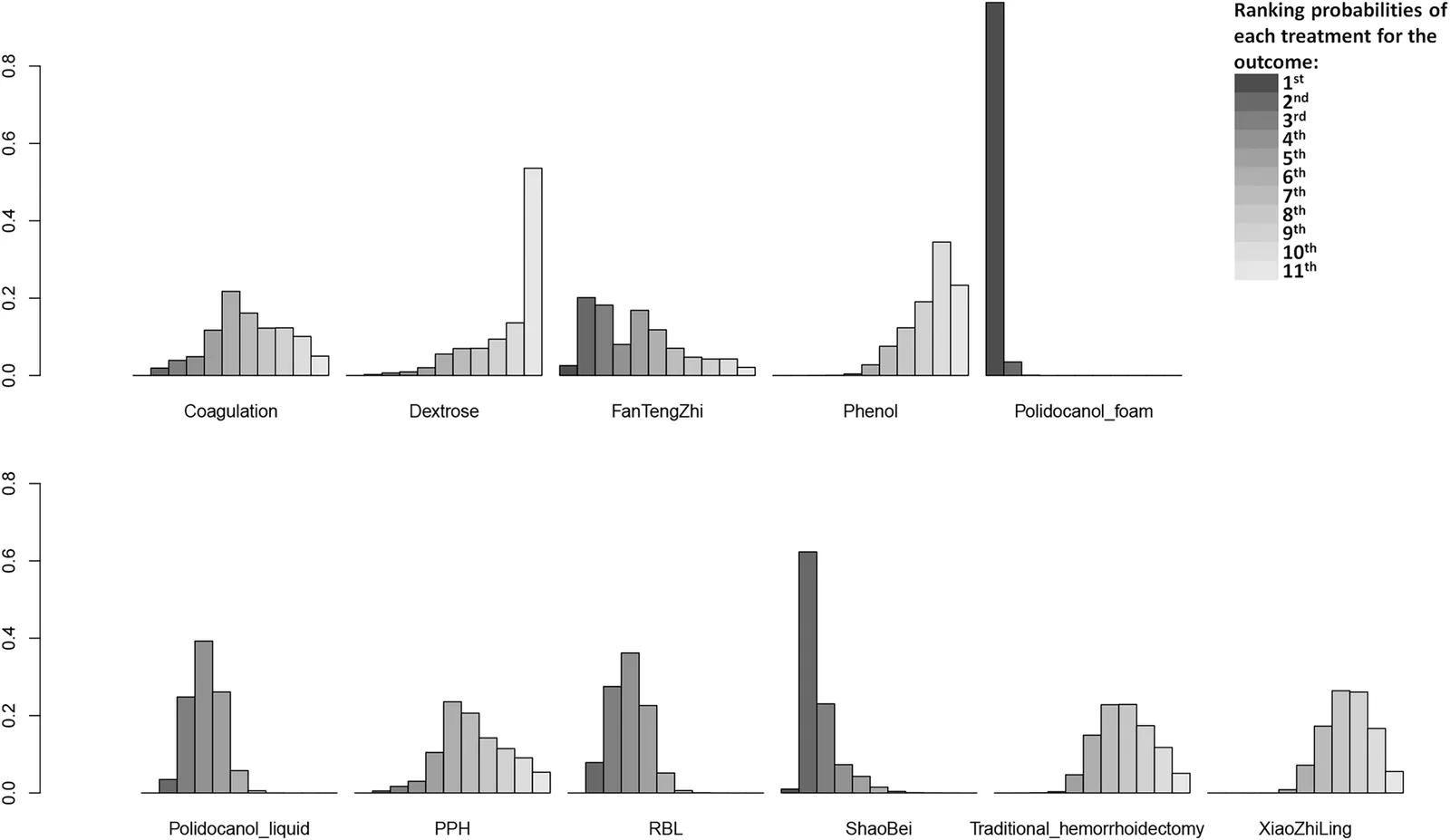
Rankogram of treatment rankings for cure rate. The graph shows the probability of each treatment occupying each possible rank (1st through 11th), based on surface under the cumulative ranking curve (SUCRA) values. PPH, procedure for prolapse and hemorrhoids; RBL, rubber band ligation.

**Table 1:** Odds ratio and 95% CI of the cure rate for each treatment on the first row as compared with each treatment on the first column.

Sclerosing agent	Polidocanol foam	Dextrose	ShaoBei	XiaoZhiLing	FanTengZhi	Phenol	Coagulation	Traditional hemorrhoidectomy	PPH	RBL
Polidocanol liquid	3.13 (2.31, 4.24)	0.35 (0.12, 0.99)	1.44 (0.78, 2.63)	0.49 (0.35, 0.7)	0.94 (0.31, 3.08)	0.42 (0.24, 0.72)	0.6 (0.26, 1.37)	0.53 (0.35, 0.82)	0.59 (0.32, 1.09)	1.02 (0.74, 1.42)
Polidocanol foam		0.11 (0.04, 0.32)	0.46 (0.24, 0.89)	0.16 (0.1, 0.24)	0.3 (0.1, 1.01)	0.13 (0.07, 0.24)	0.19 (0.08, 0.45)	0.17 (0.1, 0.29)	0.19 (0.1, 0.37)	0.33 (0.22, 0.48)
Dextrose			4.11 (1.31, 12.83)	1.41 (0.51, 3.92)	2.69 (0.75, 10.33)	1.19 (0.41, 3.46)	1.73 (0.5, 5.93)	1.52 (0.52, 4.6)	1.68 (0.52, 5.63)	2.91 (1.04, 8.34)
ShaoBei				0.34 (0.21, 0.56)	0.66 (0.2, 2.21)	0.29 (0.13, 0.64)	0.42 (0.16, 1.13)	0.37 (0.19, 0.76)	0.41 (0.18, 0.94)	0.71 (0.37, 1.39)
XiaoZhiLing					1.91 (0.67, 5.9)	0.84 (0.45, 1.56)	1.22 (0.51, 2.9)	1.08 (0.66, 1.8)	1.2 (0.61, 2.35)	2.07 (1.33, 3.22)
FanTengZhi						0.44 (0.13, 1.49)	0.64 (0.15, 2.51)	0.56 (0.16, 1.85)	0.63 (0.17, 2.23)	1.08 (0.33, 3.38)
Phenol							1.46 (0.73, 2.82)	1.29 (0.67, 2.47)	1.43 (0.63, 3.12)	2.45 (1.49, 3.99)
Coagulation								0.88 (0.36, 2.18)	0.98 (0.36, 2.66)	1.68 (0.78, 3.75)
Traditional hemorrhoidectomy									1.11 (0.53, 2.32)	1.92 (1.15, 3.14)
PPH										1.72 (0.89, 3.32)

CI, confidence interval; PPH, procedure for prolapse and hemorrhoids; RBL, rubber band ligation.

Global inconsistency was assessed by comparing the consistency and inconsistency models. The deviance information criterion (DIC) for the consistency model was 243.30, while that for the inconsistency model was 252.53, with a DIC difference of 9.23 (>5), indicating that the consistency model was substantially better than the inconsistency model. Node-splitting analysis showed that all *p*-values were greater than the conventional significance threshold (0.05), suggesting no statistically significant conflict between direct and indirect evidence. Collectively, these findings provide no evidence of global inconsistency, supporting the reliability of the consistency model results.

Heterogeneity analysis revealed an *I*^2^ of 41.5% from pairwise meta-analyses and a consistency model-derived *I*^2^ of 34.7%, indicating low to moderate heterogeneity. Two comparisons exhibited relatively high heterogeneity: coagulation vs. phenol (*I*^2^ = 75%) and polidocanol liquid vs. RBL (*I*^2^ = 75%). All other comparisons showed low heterogeneity. Importantly, the inconsistency *p*-values for all comparisons were far above 0.05 (many approaching 0.5–0.9), indicating no statistically significant inconsistency. Overall, the random-effects model was deemed appropriate, although results for comparisons with high heterogeneity should be interpreted with caution.

### Recurrence rate

The network meta-analysis results for recurrence rate are presented in Supplementary Figure S2 and Supplementary Table S2. FanTengZhi ranked as the treatment with the lowest probability of recurrence. However, caution is warranted when interpreting this finding, as the OR of comparing FanTengZhi with polidocanol liquid was extremely low (Supplementary Figure S2B), and the network plot showed that FanTengZhi was directly connected to only two other interventions. Polidocanol liquid and polidocanol foam ranked second and third lowest, respectively, in terms of recurrence probability. In contrast, 50% dextrose had the highest probability of recurrence, indicating it was the least favorable treatment for preventing recurrence.

### Patient satisfaction rate

The network meta-analysis results for patient satisfaction rate are shown in Supplementary Figure S3 and Supplementary Table S3. Polidocanol foam ranked as the treatment with the highest probability of achieving patient satisfaction, with an OR of 1.9 (95% CI: 0.98–3.7) compared with polidocanol liquid. All other treatments had odds ratios below 1 when compared with polidocanol liquid, indicating lower patient satisfaction.

### Operative complications

The network meta-analysis results for operative complications are presented in Supplementary Figure S4 and Supplementary Table S4. Among all treatments, traditional hemorrhoidectomy had the highest risk of complications, with an OR of 6.0 (95% CI: 2.8–14) compared with polidocanol liquid. Among sclerosing agents, XiaoZhiLing had the highest complication risk, with an OR of 2.7 (95% CI: 1.3–6.0) relative to polidocanol liquid. 50% dextrose and phenol showed similar complication risks to polidocanol liquid, with odds ratios close to 1. Polidocanol foam had the lowest risk of complications among all treatments, with an odds ratio of 0.53 (95% CI: 0.22–1.3) compared with polidocanol liquid, although the difference was not statistically significant. FanTengZhi ranked second lowest in complication risk.

We further analyzed five common types of operative complications: pain (Supplementary Figure S5 and Supplementary Table S5), bleeding (Supplementary Figure S6 and Supplementary Table S6), fever/infection/abscess (Supplementary Figure S7 and Supplementary Table S7), urinary retention (Supplementary Figure S8 and Supplementary Table S8), and rectal stricture (Supplementary Figure S9 and Supplementary Table S9). Polidocanol foam demonstrated significantly lower risks of bleeding (OR = 0.4, 95% CI: 0.21–0.9) and rectal stricture (OR < 0.001, 95% CI: <0.001–0.73) compared with polidocanol liquid. It also showed a trend toward lower risks of pain (OR = 0.53, 95% CI: 0.22–1.3), fever/infection/abscess (OR = 0.12, 95% CI: 0.003–1.8), and urinary retention (OR = 0.15, 95% CI: 0.0012–5). FanTengZhi also showed a trend toward reduced risks of pain and fever/infection/abscess compared with polidocanol liquid, although other complications were not reported. In contrast, XiaoZhiLing was associated with higher risks of pain and bleeding, and 50% dextrose showed a higher risk of fever/infection/abscess.

### Subgroup analyses

We performed subgroup analyses on cure rate across different patient populations, including patients with internal hemorrhoids (Supplementary Figure S10 and Supplementary Table S10), grade I/II hemorrhoids (Supplementary Figure S11 and Supplementary Table S11), and grade I/II/III hemorrhoids (Supplementary Figure S12 and Supplementary Table S12). The results consistently supported that polidocanol foam had the highest and statistically significant odds ratios on cure rate compared with polidocanol liquid across all three subgroups: internal hemorrhoids (OR = 2.9, 95% CI: 2.1–4.0), grade I/II hemorrhoids (OR = 2.7, 95% CI: 1.2–6.0), and grade I/II/III hemorrhoids (OR = 3.0, 95% CI: 1.9–4.6). Polidocanol foam had the highest probability of being the best treatment (rank 1) in all three subgroups. No other treatment showed a statistically significant advantage over polidocanol liquid in terms of cure rate in any of the subgroups.

## Discussion

Our findings demonstrate that polidocanol foam consistently ranked first in both cure rate and patient satisfaction, and exhibited a statistically significant superiority over polidocanol liquid. Regarding safety, polidocanol foam showed the lowest risk of overall complications among all treatments and was associated with significantly reduced risks of bleeding and rectal stricture compared with polidocanol liquid. In contrast, XiaoZhiLing, phenol, and 50% dextrose were significantly inferior to polidocanol liquid in terms of cure rate. Collectively, these results provide robust evidence that the foam formulation confers tangible advantages over its liquid counterpart across multiple clinically relevant domains. This study is the first to establish the comprehensive superiority of polidocanol foam for the treatment of hemorrhoids within a network meta-analytic framework.

The superiority of polidocanol foam over polidocanol liquid was consistently substantiated across multiple outcomes in our NMA. Specifically, polidocanol foam ranked first in cure rate, patient satisfaction, and low complication risk, and specifically associated with significantly lower risks of bleeding and rectal stricture. These compelling differences suggest that the foam formulation itself confers remarkable clinical advantages. From a mechanistic perspective, several physicochemical properties of foam may explain this enhanced performance. For instance, the microbubble architecture of foam enables it to fully conform to the irregular submucosal spaces of hemorrhoidal cushions, achieving a more uniform and extensive contact area with the target tissue (10, 21, 22); whereas liquid formulation tends to pool and disperse rapidly from the injection site. Foam sclerotherapy also permits the use of a lower concentration of the active sclerosant while maintaining an effective surface-active concentration, thereby reducing unintended chemical injury to adjacent mucosa. Moreover, the higher viscosity of foam prolongs its residence time at the injection site, which may result in sustained fibrotic action and more durable obliteration of hemorrhoidal veins. From a pharmacological standpoint, sclerosing agents used for hemorrhoids act through two principal mechanisms: induction of intravascular thrombosis, tissue necrosis, or a combination of both. A predominantly thrombotic mechanism is generally considered to be associated with fewer complications and a more favorable final outcome, because it produces controlled venous obliteration with limited damage to the surrounding mucosa, whereas agents whose action relies largely on tissue necrosis tend to cause deeper and less predictable injury, with greater risk of ulceration, bleeding, and stricture formation. This mechanistic distinction may, at least in part, explain the apparently better results observed with polidocanol foam. As a surface-active agent, polidocanol primarily induces endothelial damage and targeted thrombosis rather than extensive tissue necrosis, and its foam formulation further confines the sclerosant to the submucosal plane, thereby limiting necrotic depth. In contrast, agents with a predominantly necrotic mechanism, such as XiaoZhiLing, produce disproportionate tissue destruction, which is consistent with our finding that XiaoZhiLing carried the highest complication risk among sclerosing agents. Likewise, the transient hyperosmolar dehydration induced by 50% dextrose is unlikely to achieve durable thrombotic obliteration, which may account for its high recurrence probability. Collectively, these observations suggest that the efficacy and safety profile of a sclerosing agent depends not only on its physicochemical behavior but also on the balance between thrombotic and necrotic mechanisms.

Our NMA revealed that several sclerosing agents, including XiaoZhiLing, phenol, and 50% dextrose, were significantly inferior to polidocanol liquid in terms of cure rate. The divergent efficacy profiles among these agents might be mechanistically explained by their distinct pharmacologic actions and safety constraints. XiaoZhiLing, a traditional Chinese medicine-based compound typically containing alum and gallic acid derivatives, exerts its sclerosing effect through intense protein coagulation and corrosive tissue destruction (18, 23, 24). Although this mechanism induces robust fibrosis, it often leads to disproportionate tissue necrosis, ulceration, and secondary bleeding, which aligns precisely with our finding that XiaoZhiLing carried the highest complication risk among sclerosing agents (25–27). The narrow therapeutic window of XiaoZhiLing thus translates into an unfavorable balance of cure rate and adverse event. Phenol, a classic sclerosing agent, is limited by its well-documented cardiac and neurotoxicity, which restricts clinical use to low concentrations (typically <5%). The modest fibrotic effect at such concentrations likely explains its suboptimal cure rate, as the induced inflammatory response may be insufficient to achieve complete and durable involution of hemorrhoidal cushions (28). In contrast, 50% dextrose operates via a fundamentally distinct mechanism with hyperosmolar dehydration rather than surface-active protein denaturation. This effect is inherently transient, resulting in high recurrence rates, which is corroborated by our ranking analysis showing that 50% dextrose had the highest probability of recurrence. Collectively, these findings underscore that the efficacy of sclerotherapy is critically dependent not merely on the presence of a sclerosing effect, but on the agent's ability to deliver a targeted, durable fibrotic response.

Beyond overall complications, our NMA revealed distinct safety profiles across interventions with important clinical implications. Polidocanol foam significantly reduced the risks of bleeding and rectal stricture compared with polidocanol liquid. These advantages are mainly attributed to the foam's controlled submucosal deposition, which limits necrotic depth and avoids the transmural injury commonly associated with liquid sclerosants, thereby lowering stricture risk (29, 30). The reduced bleeding risk further reflects foam's ability to induce targeted thrombosis without widespread mucosal ulceration (31). Regarding postoperative pain, conventional hemorrhoidectomy carried the highest risk, reinforcing guideline recommendations that office-based, minimally invasive procedures should be the first-line approach for grade I–III hemorrhoids (32). Nevertheless, while current guidelines generally state that polidocanol is effective without specifying formulation, our results provide the first network meta-analytic evidence that polidocanol foam is significantly superior to its liquid counterpart across multiple outcomes. This refinement carries important implications for future guideline updates toward formulation-specific recommendations. The robustness of this advantage is further underscored by our subgroup analyses, which consistently demonstrated the superiority of polidocanol foam across populations with internal hemorrhoids, grade I/II hemorrhoids, and grade I/II/III hemorrhoids, respectively. This consistency simplifies clinical decision-making by supporting polidocanol foam as a reasonable first-choice sclerosing agent across a broad range of patients. Conversely, our results do not suggest the using of phenol in clinical practice, given its significantly inferior cure rate and notable toxicity.

Two comparisons exhibited substantial heterogeneity (*I*^2^ = 75%): coagulation vs. phenol and polidocanol liquid vs. RBL. Several clinical factors may account for this variability. First, injection technique varied considerably across trials, including differences in injection volume, depth (submucosal vs. deeper injection), number and spacing of injection sites, and the use of endoscopic vs. anoscopic guidance, all of which can influence the extent of fibrotic response and thereby cure rates. Second, drug concentration and formulation were not uniform: phenol was used at concentrations typically below 5% but with differing oil or aqueous vehicles, while polidocanol liquid was administered at varying concentrations, and the total dose delivered per session differed across studies. Third, follow-up durations ranged from several weeks to more than 1 year, which may differentially capture early treatment success vs. late recurrence and thus contribute to between-study variance. Furthermore, the comparator procedures themselves are operator-dependent; RBL outcomes are influenced by the number of bands applied, the ligation device used, and the experience of the endoscopist, while coagulation may be performed with different energy settings and devices. Given that the node-splitting analysis showed no significant inconsistency between direct and indirect evidence, these high-heterogeneity comparisons likely reflect genuine clinical and methodological diversity rather than violation of the consistency assumption. Nonetheless, the estimates for these two comparisons should be interpreted with caution, and future trials should report injection parameters, drug concentrations, and follow-up schedules in a standardized manner to reduce heterogeneity.

Several strengths of this NMA warrant mention. The consistency model demonstrated superior fit over the inconsistency model, and node-splitting analysis yielded no significant conflicts between direct and indirect evidence, collectively supporting to the robustness and reliability of the NMA. Nevertheless, several limitations must be acknowledged. First, some comparisons yielded extreme effect estimates and wide confidence intervals owing to zero events or sparse data. For instance, the risk of rectal stricture with polidocanol foam was estimated as OR < 0.001 (95% CI: <0.001–0.73), and comparisons involving FanTengZhi or 50% dextrose were based on few events and small sample sizes, resulting in imprecise estimates. Such extreme values are typical artifacts of zero-event or sparse-data comparisons in network meta-analysis rather than genuine estimates of infinite or negligible effect, and they should therefore be interpreted with caution; continuity corrections and sensitivity analyses may be warranted in future updates as more evidence accumulates. Second, the evidence base for recurrence rate was less meaningful than that for cure rate, as most included studies had follow-up durations of less than 1 year, potentially underestimating true recurrence rates. Consequently, the long-term efficacy of polidocanol foam remains to be established in future studies with extended follow-up.

## Conclusion

This network meta-analysis demonstrates that polidocanol foam is superior to polidocanol liquid and other sclerosing agents for treating grade I–III internal hemorrhoids, ranking first in cure rate and patient satisfaction while showing the lowest complication risks, particularly for bleeding and rectal stricture. These findings support polidocanol foam as the preferred sclerosing agent and advocate for formulation-specific considerations in clinical practice. Nevertheless, given that most included trials followed patients for less than 1 year, these conclusions pertain primarily to short- and mid-term outcomes, and the long-term durability awaits confirmation in trials with extended follow-up.

## Data Availability

The raw data supporting the conclusions of this article will be made available by the authors, without undue reservation.
