# Supplementary material for: Clinical outcomes of different sclerosing agents for hemorrhoids: a network meta-analysis
Source: Front Med (Lausanne). 2026 Oct 7;13:1915729. doi: 10.3389/fmed.2026.1915729 (PMC13683147; doi:10.3389/fmed.2026.1915729)
Supplement: Supplementary file 1 [file Table1.docx]

**Supplementary Materials**

**Composition the traditional Chinese medicine-based sclerosing agents**

**XiaoZhiLing:** Alum, tannic acid, chlorobutanol, low-molecular-weight dextran, sodium citrate, sodium bisulfite, glycerol. Alum and tannic acid are considered the principal sclerosing components, inducing protein coagulation and fibrotic tissue reaction; chlorobutanol acts as a preservative, and the remaining agents serve as stabilizers, osmolarity adjusters, or vehicles.

**ShaoBei**: Citric acid, gallic acid, paeoniflorin. Gallic acid and paeoniflorin are regarded as the main active constituents, contributing to local inflammatory and fibrotic responses; citric acid serves as an acidity regulator.

**FanTengZhi**: Alum, palmatine, halloysite/red halloysite. Alum and palmatine are considered the principal active components responsible for the sclerosing and anti-inflammatory effects; halloysite acts as a carrier/excipient.

**Table S1. Characteristics and quality assessments of included studies**

| Study | Internal, external, or mixed hemorrhoids | Hemorrhagic hemorrhoids | Grade of hemorrhoid | Bias arising from the randomization process | Bias due to deviations from intended interventions | Bias due to missing outcome data | Bias in measurement of the outcome | Bias in selection of the reported result | Treatment | Group sample size |
| --- | --- | --- | --- | --- | --- | --- | --- | --- | --- | --- |
| Varma et al. 1991(1) | Internal | Unrestricted | I/II | Low risk | Some concerns | Low risk | Some concerns | Low risk | Phenol | 28 |
|  |  |  |  |  |  |  |  |  | Coagulation | 23 |
| Walker et al. 1990(2) | Internal | Unrestricted | I/II | Low risk | Some concerns | Low risk | Low risk | Low risk | Phenol | 60 |
|  |  |  |  |  |  |  |  |  | RBL | 40 |
|  |  |  |  |  |  |  |  |  | Coagulation | 100 |
| Ambrose et al. 1985(3) | Internal | Unrestricted | I/II | Some concerns | Low risk | Low risk | Low risk | Low risk | Phenol | 62 |
|  |  |  |  |  |  |  |  |  | Coagulation | 73 |
| Gartell et al., 1985(4) | Internal | Unrestricted | I/II/III/IV | Some concerns | Low risk | Some concerns | Low risk | Low risk | Phenol | 134 |
|  |  |  |  |  |  |  |  |  | RBL | 135 |
| Cheng et al. 1981(5) | Internal | Unrestricted | II | Some concerns | Low risk | Low risk | Low risk | Low risk | Phenol | 30 |
|  |  |  |  |  |  |  |  |  | RBL | 30 |
|  |  |  |  |  |  |  |  |  | TH | 30 |
| Greca et al. 1981(6) | Internal | Unrestricted | I/II/III | Low risk | Low risk | Some concerns | Low risk | Low risk | Phenol | 43 |
|  |  |  |  |  |  |  |  |  | RBL | 39 |
| Kanellos et al. 2002(7) | Internal | Unrestricted | II | Some concerns | Low risk | Low risk | Low risk | Low risk | Phenol | 85 |
|  |  |  |  |  |  |  |  |  | RBL | 85 |
| Moser et al. 2013(8) | Internal | Yes | I | Low risk | Low risk | Low risk | Low risk | Low risk | Polidocanol liquid | 64 |
|  |  |  |  |  |  |  |  |  | Polidocanol foam | 66 |
| Akindiose et al. 2016(9) | Internal | Unrestricted | I/II/III | Low risk | Low risk | Low risk | Low risk | Low risk | Phenol | 40 |
|  |  |  |  |  |  |  |  |  | 50% dextrose | 40 |
| Abiodun et al. 2020(10) | Internal | Unrestricted | II/III | Low risk | Low risk | Low risk | Low risk | Low risk | 50% dextrose | 30 |
|  |  |  |  |  |  |  |  |  | RBL | 30 |
| Mishra et al. 2020(11) | Internal | Yes | I/II | Low risk | Low risk | Low risk | Low risk | Low risk | Polidocanol liquid | 75 |
|  |  |  |  |  |  |  |  |  | Phenol | 75 |
| Salgueiro et al. 2022(12) | Internal | Unrestricted | I/II/III | Low risk | Low risk | Low risk | Low risk | Low risk | Polidocanol foam | 60 |
|  |  |  |  |  |  |  |  |  | RBL | 60 |
| Cestaro et al. 2013(13) | Internal | Unrestricted | II | Low risk | Low risk | Low risk | Low risk | Low risk | Polidocanol liquid | 36 |
|  |  |  |  |  |  |  |  |  | RBL | 36 |
| Shen et al. 2019(14) | Internal | Yes | I | Low risk | Low risk | Low risk | Low risk | Low risk | Polidocanol foam | 55 |
|  |  |  |  |  |  |  |  |  | Polidocanol liquid | 53 |
| Xin 2025(15) | Internal | Yes | Unreported | Some concerns | Low risk | Low risk | Low risk | Low risk | Polidocanol liquid | 40 |
|  |  |  |  |  |  |  |  |  | TH | 40 |
| Cai et al. 2022(16) | Internal | Unrestricted | I/II/III | Some concerns | Low risk | Low risk | Low risk | Low risk | Polidocanol foam | 47 |
|  |  |  |  |  |  |  |  |  | Polidocanol liquid | 47 |
| Zhang et al. 2018(17) | Mixed | Unrestricted | Unreported | Some concerns | Low risk | Low risk | Low risk | Low risk | FanTengZhi | 30 |
|  |  |  |  |  |  |  |  |  | XiaoZhiLing | 30 |
|  |  |  |  |  |  |  |  |  | 50% dextrose | 30 |
| Kong et al. 2022(18) | Internal | Unrestricted | I/II | Low risk | Low risk | Low risk | Low risk | Low risk | FanTengZhi | 35 |
|  |  |  |  |  |  |  |  |  | XiaoZhiLing | 35 |
| Liang et al. 2015(19) | Internal | Yes | I/II/III | Some concerns | Low risk | Low risk | Low risk | Low risk | Polidocanol liquid | 126 |
|  |  |  |  |  |  |  |  |  | XiaoZhiLing | 122 |
| Lian et al. 2016(20) | Internal | Unrestricted | II/III | Some concerns | Low risk | Low risk | Low risk | Low risk | XiaoZhiLing | 39 |
|  |  |  |  |  |  |  |  |  | RBL | 39 |
| Li et al. 2020(21) | Internal | Unrestricted | II/III | Some concerns | Low risk | Low risk | Low risk | Low risk | Polidocanol liquid | 30 |
|  |  |  |  |  |  |  |  |  | XiaoZhiLing | 30 |
| Liu et al. 2024(a)(22) | Internal | Unrestricted | Unreported | Low risk | Low risk | Low risk | Low risk | Low risk | Polidocanol liquid | 190 |
|  |  |  |  |  |  |  |  |  | XiaoZhiLing | 190 |
| Lin et al. 2021(23) | Internal | Unrestricted | I/II/III | Some concerns | Low risk | Low risk | Low risk | Low risk | Polidocanol liquid | 40 |
|  |  |  |  |  |  |  |  |  | TH | 40 |
| Huo et al. 2022(24) | Mixed | Unrestricted | II/III | Low risk | Low risk | Low risk | Low risk | Low risk | XiaoZhiLing | 30 |
|  |  |  |  |  |  |  |  |  | TH | 30 |
| Tan et al. 2018(25) | Internal | Unrestricted | Unreported | Some concerns | Low risk | Low risk | Low risk | Low risk | Polidocanol liquid | 60 |
|  |  |  | Unreported |  |  |  |  |  | PPH | 60 |
| Liu et al. 2024(b)(26) | Mixed | Unrestricted | I/II/III/IV | Low risk | Low risk | Low risk | Low risk | Low risk | Polidocanol foam | 57 |
|  |  |  |  |  |  |  |  |  | Polidocanol liquid | 57 |
| Li et al. 2025(27) | Internal | Unrestricted | I/II/III | Some concerns | Low risk | Low risk | Low risk | Low risk | Polidocanol foam | 64 |
|  |  |  |  |  |  |  |  |  | RBL | 64 |
| Ai et al. 2020(28) | Internal | Yes | Unreported | Low risk | Low risk | Low risk | Some concerns | Low risk | Polidocanol liquid | 105 |
|  |  |  |  |  |  |  |  |  | Polidocanol foam | 105 |
| Tang et al. 2021(a)(29) | Internal | Yes | I/II/III | Some concerns | Low risk | Low risk | Low risk | Low risk | Polidocanol liquid | 51 |
|  |  |  |  |  |  |  |  |  | Polidocanol foam | 52 |
| Ai et al. 2021(30) | Internal | Yes | Unreported | Some concerns | Low risk | Low risk | Low risk | Low risk | Polidocanol liquid | 111 |
|  |  |  |  |  |  |  |  |  | XiaoZhiLing | 102 |
| Lei et al. 2017(31) | Internal | Yes | I/II | Some concerns | Low risk | Low risk | Low risk | Low risk | Polidocanol liquid | 94 |
|  |  |  |  |  |  |  |  |  | XiaoZhiLing | 88 |
| Liu 2022(32) | Internal | Yes | Unreported | Some concerns | Low risk | Low risk | Low risk | Low risk | Polidocanol liquid | 39 |
|  |  |  |  |  |  |  |  |  | XiaoZhiLing | 39 |
| Wang et al. 2018(33) | Internal | Yes | I | Some concerns | Low risk | Low risk | Low risk | Low risk | Polidocanol liquid | 64 |
|  |  |  |  |  |  |  |  |  | Polidocanol foam | 66 |
| Xu et al. 2014(34) | Internal | Unrestricted | Unreported | Some concerns | Low risk | Low risk | Low risk | Low risk | Polidocanol liquid | 60 |
|  |  |  |  |  |  |  |  |  | XiaoZhiLing | 60 |
| Zhang et al. 2019(35) | Internal | Yes | I/II | Some concerns | Low risk | Low risk | Low risk | Low risk | Polidocanol liquid | 45 |
|  |  |  |  |  |  |  |  |  | XiaoZhiLing | 45 |
| Dong et al. 2025(36) | Internal | Yes | II/III | Some concerns | Low risk | Low risk | Low risk | Low risk | Polidocanol liquid | 32 |
|  |  |  |  |  |  |  |  |  | RBL | 32 |
| Chen et al. 2023(a)(37) | Internal | Unrestricted | II/III | Low risk | Low risk | Low risk | Low risk | Low risk | Polidocanol liquid | 60 |
|  |  |  |  |  |  |  |  |  | PPH | 60 |
| Tang et al. 2021(b)(38) | Internal | Unrestricted | Unreported | Some concerns | Low risk | Low risk | Low risk | Low risk | Polidocanol foam | 50 |
|  |  |  |  |  |  |  |  |  | XiaoZhiLing | 50 |
| Xiao et al. 2023(39) | Internal | Yes | II | Low risk | Low risk | Low risk | Low risk | Low risk | Polidocanol liquid | 41 |
|  |  |  |  |  |  |  |  |  | Polidocanol foam | 40 |
| Zhang et al. 2023(40) | Internal | Unrestricted | I/II/III | Low risk | Low risk | Low risk | Low risk | Low risk | Polidocanol foam | 56 |
|  |  |  |  |  |  |  |  |  | Polidocanol liquid | 52 |
| Wei et al. 2023(41) | Internal | Unrestricted | II/III | Some concerns | Low risk | Low risk | Low risk | Low risk | XiaoZhiLing | 31 |
|  |  |  |  |  |  |  |  |  | RBL | 31 |
|  |  |  |  |  |  |  |  |  | PPH | 31 |
| Ye et al. 2022(42) | Internal | Unrestricted | I/II/III | Low risk | Low risk | Low risk | Low risk | Low risk | Polidocanol foam | 50 |
|  |  |  |  |  |  |  |  |  | Polidocanol liquid | 50 |
|  |  |  |  |  |  |  |  |  | RBL | 50 |
| Lu et al. 2024(43) | Internal | Unrestricted | II/III | Low risk | Low risk | Low risk | Low risk | Low risk | Polidocanol liquid | 32 |
|  |  |  |  |  |  |  |  |  | RBL | 32 |
| Chen et al. 2022(44) | Internal | Unrestricted | II/III | Low risk | Low risk | Low risk | Low risk | Low risk | Polidocanol liquid | 33 |
|  |  |  |  |  |  |  |  |  | RBL | 33 |
| Che 2024(45) | Internal | Unrestricted | II | Low risk | Low risk | Low risk | Low risk | Low risk | Polidocanol liquid | 40 |
|  |  |  |  |  |  |  |  |  | RBL | 40 |
| Zhong et al. 2022(46) | Unrestricted | Unrestricted | I/II/III | Low risk | Low risk | Low risk | Low risk | Low risk | Polidocanol foam | 27 |
|  |  |  |  |  |  |  |  |  | RBL | 26 |
| Yu et al. 2023(47) | Internal | Unrestricted | I/II/III | Some concerns | Low risk | Low risk | Low risk | Low risk | XiaoZhiLing | 60 |
|  |  |  |  |  |  |  |  |  | TH | 60 |
| LYU et al. 2021(48) | Internal | Unrestricted | I/II/III | Low risk | Low risk | Low risk | Low risk | Low risk | Polidocanol liquid | 40 |
|  |  |  |  |  |  |  |  |  | TH | 40 |
| Yuan et al. 2023(49) | Internal | Unrestricted | II/III | Low risk | Low risk | Low risk | Low risk | Low risk | Polidocanol liquid | 46 |
|  |  |  |  |  |  |  |  |  | RBL | 49 |
| Ren et al. 2023(50) | Internal | Unrestricted | I/II/III | Some concerns | Low risk | Low risk | Low risk | Low risk | Polidocanol liquid | 30 |
|  |  |  |  |  |  |  |  |  | PPH | 30 |
| Chen et al. 2021(51) | Internal | Unrestricted | Unreported | Low risk | Low risk | Low risk | Low risk | Low risk | Polidocanol liquid | 40 |
|  |  |  |  |  |  |  |  |  | Polidocanol foam | 40 |
| Feng et al. 2016(52) | Mixed | Unrestricted | Unreported | Low risk | Low risk | Low risk | Low risk | Low risk | XiaoZhiLing | 57 |
|  |  |  |  |  |  |  |  |  | ShaoBei | 68 |
| Wang et al. 2013(53) | Internal | Unrestricted | II/III | Some concerns | Low risk | Low risk | Low risk | Low risk | XiaoZhiLing | 80 |
|  |  |  |  |  |  |  |  |  | ShaoBei | 80 |
| Wang et al. 2008(54) | Internal/mixed | Unrestricted | Unreported | Some concerns | Low risk | Low risk | Low risk | Low risk | ShaoBei | 216 |
|  |  |  |  |  |  |  |  |  | XiaoZhiLing | 198 |
| Liu et al. 2008(55) | Internal/mixed | Unrestricted | Unreported | High risk | Low risk | Low risk | Low risk | Low risk | XiaoZhiLing | 30 |
|  |  |  |  |  |  |  |  |  | ShaoBei | 32 |
| Liao et al. 2008(56) | Internal/mixed | Unrestricted | I/II/III | Some concerns | Low risk | Low risk | Low risk | Low risk | XiaoZhiLing | 120 |
|  |  |  |  |  |  |  |  |  | ShaoBei | 385 |
| Chen et al. 2023(b)(57) | Internal | Yes | Unreported | Some concerns | Low risk | Low risk | Low risk | Low risk | Polidocanol liquid | 34 |
|  |  |  |  |  |  |  |  |  | TH | 34 |
| Gui et al. 2024(58) | Internal | Unrestricted | I/II/III | Low risk | Low risk | Low risk | Low risk | Low risk | Polidocanol foam | 40 |
|  |  |  |  |  |  |  |  |  | RBL | 40 |
| Huang et al. 2020(59) | Internal | Unrestricted | I/II/III | Some concerns | Low risk | Low risk | Low risk | Low risk | Polidocanol foam | 195 |
|  |  |  |  |  |  |  |  |  | Polidocanol liquid | 151 |
| Yan et al. 2025(60) | Internal | Unrestricted | II/III | Low risk | Low risk | Low risk | Low risk | Low risk | Polidocanol foam | 39 |
|  |  |  |  |  |  |  |  |  | RBL | 39 |
| Xiong et al. 2022(61) | Unrestricted | Unrestricted | Unreported | Low risk | Low risk | Low risk | Low risk | Low risk | Polidocanol liquid | 60 |
|  |  |  |  |  |  |  |  |  | TH | 60 |
| Zheng et al. 2024(62) | Internal | Unrestricted | II/III | Low risk | Low risk | Low risk | Low risk | Low risk | Polidocanol liquid | 35 |
|  |  |  |  |  |  |  |  |  | RBL | 35 |
| Zhou et al. 2021(a)(63) | Unrestricted | Unrestricted | Unreported | Some concerns | Low risk | Low risk | Low risk | Low risk | Polidocanol liquid | 52 |
|  |  |  |  |  |  |  |  |  | TH | 52 |
| Zhou et al. 2021(b)(64) | Internal | Unrestricted | I/II/III | Low risk | Low risk | Low risk | Low risk | Low risk | Polidocanol liquid | 69 |
|  |  |  |  |  |  |  |  |  | RBL | 66 |
| Zhao et al. 2022(65) | Internal | Yes | I/II/III | Some concerns | Low risk | Low risk | Low risk | Low risk | Polidocanol foam | 25 |
|  |  |  |  |  |  |  |  |  | Polidocanol liquid | 25 |
| Ni et al. 2020(66) | Internal | Unrestricted | Unreported | Some concerns | Low risk | Low risk | Low risk | Low risk | Polidocanol liquid | 39 |
|  |  |  |  |  |  |  |  |  | XiaoZhiLing | 39 |

The grade of internal hemorrhoids was classified according to the Goligher classification or referring to the Chinese Guidelines for the Diagnosis and Treatment of Hemorrhoids (2010 Revision). RBL, rubber band ligation; PPH, procedure for prolapse and hemorrhoids; TH, traditional hemorrhoidectomy.

**Table S2. Odds ratio and 95% CI of the recurrence rate for each treatment on the first row (red color) as compared with each treatment on the first column (green color)**

|  | Polidocanol foam | Dextrose | XiaoZhiLing | FanTengZhi | Phenol | Coagulation | Traditional hemorrhoidectomy | PPH | RBL |
| --- | --- | --- | --- | --- | --- | --- | --- | --- | --- |
| Polidocanol liquid | 1.14 (0.42, 3.96) | 22.06 (3.33, 220.23) | 5.91 (2.13, 23.11) | 0 (0, 0.48) | 9.14 (2.61, 40.99) | 10.6 (1.88, 82.48) | 3.52 (0.88, 16.67) | 2.23 (0.21, 33.9) | 3.51 (1.68, 8.81) |
| Polidocanol foam |  | 19.1 (2.26, 212.5) | 5.18 (1.21, 25.5) | 0 (0, 0.43) | 8.03 (1.88, 34.41) | 9.4 (1.34, 68) | 3.08 (0.55, 17.31) | 1.93 (0.13, 35.59) | 3.09 (1.19, 7.77) |
| Dextrose |  |  | 0.27 (0.03, 1.82) | 0 (0, 0.02) | 0.42 (0.04, 3.96) | 0.49 (0.03, 6.56) | 0.16 (0.01, 1.73) | 0.1 (0, 2.6) | 0.16 (0.02, 1.1) |
| XiaoZhiLing |  |  |  | 0 (0, 0.08) | 1.55 (0.27, 8.19) | 1.82 (0.21, 14.37) | 0.6 (0.09, 3.49) | 0.37 (0.02, 6.29) | 0.6 (0.15, 2.02) |
| FanTengZhi |  |  |  |  | 3.8364703471444e+19 (17.53, 6.20621117170702e+43) | 46413879975231700992 (20.02, 9.11842809283473e+43) | 14708815456530407424 (7.06, 1.84936654386068e+43) | 9420783975652802560 (4.29, 1.699104572329e+43) | 14288069333203392512 (7.4, 2.1666161819755e+43) |
| Phenol |  |  |  |  |  | 1.18 (0.21, 6.29) | 0.39 (0.07, 2.04) | 0.25 (0.01, 4.75) | 0.39 (0.12, 1.15) |
| Coagulation |  |  |  |  |  |  | 0.33 (0.03, 2.98) | 0.21 (0.01, 5.31) | 0.33 (0.06, 1.77) |
| Traditional hemorrhoidectomy |  |  |  |  |  |  |  | 0.63 (0.04, 12.85) | 1 (0.22, 4.51) |
| PPH |  |  |  |  |  |  |  |  | 1.59 (0.1, 20.73) |

CI: confidence interval; PPH, procedure for prolapse and hemorrhoids; RBL, rubber band ligation.

**Table S3. Odds ratio and 95% CI of the satisfaction rate for each treatment on the first row (red color) as compared with each treatment on the first column (green color)**

|  | Polidocanol foam | Dextrose | XiaoZhiLing | Phenol | Coagulation | Traditional hemorrhoidectomy | RBL |
| --- | --- | --- | --- | --- | --- | --- | --- |
| Polidocanol liquid | 1.85 (0.98, 3.68) | 0.27 (0.05, 1.24) | 0.17 (0.02, 0.9) | 0.29 (0.09, 0.79) | 0.48 (0.06, 3.82) | 0.44 (0.07, 2.22) | 0.3 (0.08, 0.92) |
| Polidocanol foam |  | 0.15 (0.02, 0.75) | 0.09 (0.01, 0.54) | 0.16 (0.04, 0.52) | 0.26 (0.03, 2.18) | 0.24 (0.03, 1.35) | 0.16 (0.04, 0.57) |
| Dextrose |  |  | 0.62 (0.04, 6.46) | 1.06 (0.31, 3.57) | 1.75 (0.22, 15.21) | 1.6 (0.31, 8.56) | 1.09 (0.34, 3.34) |
| XiaoZhiLing |  |  |  | 1.71 (0.21, 19.67) | 2.92 (0.19, 54.81) | 2.65 (0.22, 37.99) | 1.76 (0.21, 21.13) |
| Phenol |  |  |  |  | 1.68 (0.31, 9.9) | 1.52 (0.41, 5.73) | 1.04 (0.45, 2.31) |
| Coagulation |  |  |  |  |  | 0.91 (0.1, 7.55) | 0.61 (0.09, 3.78) |
| Traditional hemorrhoidectomy |  |  |  |  |  |  | 0.67 (0.18, 2.51) |

CI: confidence interval; RBL, rubber band ligation.

**Table S4. Odds ratio and 95% CI of the overall complication rate for each treatment on the first row (red color) as compared with each treatment on the first column (green color)**

|  | Polidocanol foam | Dextrose | XiaoZhiLing | FanTengZhi | Phenol | Coagulation | Traditional hemorrhoidectomy | PPH | RBL |
| --- | --- | --- | --- | --- | --- | --- | --- | --- | --- |
| Polidocanol liquid | 0.53 (0.22, 1.27) | 1.16 (0.27, 4.55) | 2.74 (1.27, 5.99) | 0.64 (0.08, 4.72) | 0.87 (0.26, 3.28) | 0.19 (0.03, 1.22) | 5.98 (2.79, 13.62) | 3.65 (1.17, 12.04) | 1.46 (0.75, 2.85) |
| Polidocanol foam |  | 2.2 (0.44, 10.39) | 5.18 (1.66, 16.54) | 1.2 (0.14, 10.19) | 1.64 (0.41, 7.76) | 0.35 (0.05, 2.56) | 11.31 (3.64, 37.46) | 6.98 (1.66, 29.48) | 2.75 (1.13, 6.93) |
| Dextrose |  |  | 2.37 (0.6, 10.27) | 0.55 (0.08, 3.91) | 0.74 (0.16, 4.5) | 0.16 (0.02, 1.49) | 5.16 (1.13, 26.36) | 3.17 (0.56, 19.51) | 1.26 (0.33, 5.21) |
| XiaoZhiLing |  |  |  | 0.23 (0.03, 1.61) | 0.32 (0.08, 1.37) | 0.07 (0.01, 0.48) | 2.18 (0.78, 6.44) | 1.34 (0.38, 4.91) | 0.53 (0.21, 1.36) |
| FanTengZhi |  |  |  |  | 1.35 (0.15, 14.73) | 0.3 (0.02, 4.43) | 9.31 (1.15, 86.1) | 5.76 (0.6, 60.96) | 2.28 (0.29, 18.74) |
| Phenol |  |  |  |  |  | 0.21 (0.04, 1.08) | 6.87 (1.66, 26.47) | 4.26 (0.72, 20.5) | 1.69 (0.49, 4.98) |
| Coagulation |  |  |  |  |  |  | 32.02 (4.65, 212.34) | 19.87 (2.27, 156.79) | 7.87 (1.35, 41.14) |
| Traditional hemorrhoidectomy |  |  |  |  |  |  |  | 0.62 (0.15, 2.47) | 0.24 (0.09, 0.63) |
| PPH |  |  |  |  |  |  |  |  | 0.4 (0.11, 1.41) |

CI: confidence interval; PPH, procedure for prolapse and hemorrhoids; RBL, rubber band ligation.

**Table S5. Odds ratio and 95% CI of the complication rate (pain) for each treatment on the first row (red color) as compared with each treatment on the first column (green color)**

|  | Polidocanol foam | Dextrose | XiaoZhiLing | FanTengZhi | Phenol | Coagulation | Traditional hemorrhoidectomy | PPH | RBL |
| --- | --- | --- | --- | --- | --- | --- | --- | --- | --- |
| Polidocanol liquid | 0.53 (0.22, 1.26) | 1.15 (0.28, 4.46) | 2.72 (1.25, 6.11) | 0.62 (0.07, 4.71) | 0.87 (0.26, 3.36) | 0.19 (0.03, 1.15) | 5.93 (2.75, 13.64) | 3.62 (1.15, 12) | 1.46 (0.76, 2.85) |
| Polidocanol foam |  | 2.19 (0.43, 10.46) | 5.19 (1.67, 16.28) | 1.17 (0.12, 10.26) | 1.65 (0.42, 7.54) | 0.35 (0.05, 2.52) | 11.3 (3.61, 37.35) | 6.91 (1.68, 29.29) | 2.77 (1.12, 7.05) |
| Dextrose |  |  | 2.36 (0.6, 10.29) | 0.54 (0.07, 3.81) | 0.76 (0.16, 4.63) | 0.16 (0.02, 1.51) | 5.18 (1.14, 26.96) | 3.16 (0.56, 18.99) | 1.26 (0.33, 5.13) |
| XiaoZhiLing |  |  |  | 0.22 (0.03, 1.56) | 0.32 (0.08, 1.4) | 0.07 (0.01, 0.47) | 2.17 (0.78, 6.42) | 1.33 (0.37, 4.83) | 0.53 (0.21, 1.35) |
| FanTengZhi |  |  |  |  | 1.41 (0.16, 15.5) | 0.3 (0.02, 4.54) | 9.69 (1.15, 92.97) | 5.86 (0.64, 61.51) | 2.36 (0.31, 20.17) |
| Phenol |  |  |  |  |  | 0.21 (0.04, 1.06) | 6.75 (1.61, 26.39) | 4.14 (0.72, 20.01) | 1.67 (0.49, 4.89) |
| Coagulation |  |  |  |  |  |  | 31.9 (4.62, 213.89) | 19.62 (2.35, 154.87) | 7.88 (1.41, 42.07) |
| Traditional hemorrhoidectomy |  |  |  |  |  |  |  | 0.61 (0.15, 2.38) | 0.25 (0.09, 0.63) |
| PPH |  |  |  |  |  |  |  |  | 0.4 (0.11, 1.41) |

CI: confidence interval; PPH, procedure for prolapse and hemorrhoids; RBL, rubber band ligation.

**Table S6. Odds ratio and 95% CI of the complication (bleeding) rate for each treatment on the first row (red color) as compared with each treatment on the first column (green color)**

|  | Polidocanol foam | XiaoZhiLing | Phenol | Coagulation | Traditional hemorrhoidectomy | PPH | RBL |
| --- | --- | --- | --- | --- | --- | --- | --- |
| Polidocanol liquid | 0.4 (0.21, 0.9) | 2.87 (0.43, 21.84) | 0.47 (0.08, 2.3) | 1.24 (0.24, 6.07) | 11.65 (2.59, 106.93) | 2.04 (0.54, 10.31) | 0.89 (0.36, 2.18) |
| Polidocanol foam |  | 7.14 (0.92, 54.35) | 1.17 (0.17, 5.82) | 3.13 (0.52, 15.77) | 29.23 (5.65, 280.86) | 5.2 (1.11, 27.72) | 2.23 (0.79, 5.9) |
| XiaoZhiLing |  |  | 0.16 (0.01, 1.7) | 0.44 (0.04, 4.84) | 4.21 (0.45, 64.11) | 0.72 (0.13, 4.4) | 0.31 (0.04, 2.11) |
| Phenol |  |  |  | 2.63 (0.68, 12.13) | 25.11 (3.33, 490.19) | 4.43 (0.61, 43.36) | 1.94 (0.48, 9.26) |
| Coagulation |  |  |  |  | 9.38 (1.19, 151.9) | 1.68 (0.24, 14.14) | 0.72 (0.18, 3.11) |
| Traditional hemorrhoidectomy |  |  |  |  |  | 0.18 (0.01, 1.28) | 0.07 (0.01, 0.4) |
| PPH |  |  |  |  |  |  | 0.44 (0.08, 1.81) |

CI: confidence interval; PPH, procedure for prolapse and hemorrhoids; RBL, rubber band ligation.

**Table S7. Odds ratio and 95% CI of the complication (fever, infection, or abscess) rate for each treatment on the first row (red color) as compared with each treatment on the first column (green color)**

|  | Polidocanol foam | Dextrose | XiaoZhiLing | FanTengZhi | Phenol | Traditional hemorrhoidectomy | PPH | RBL |
| --- | --- | --- | --- | --- | --- | --- | --- | --- |
| Polidocanol liquid | 0.12 (0, 1.81) | 1426734908.08 (0, 2.33625075927456e+29) | 14.49 (1.29, 448.26) | 0 (0, 3.72858094189305e+20) | 334.98 (0, 5.51746395855741e+23) | 7.59 (1.22, 103.2) | 5.69 (0.84, 58.69) | 1.42 (0.16, 10.33) |
| Polidocanol foam |  | 13133281945.03 (0, 1.76557056907137e+30) | 131.36 (3.35, 23123.85) | 0.02 (0, 3.99171254079635e+21) | 3152.8 (0, 6.2315610986965e+24) | 69.4 (2.4, 6417.13) | 52.73 (1.75, 3941.51) | 12.4 (0.41, 779.26) |
| Dextrose |  |  | 0 (0, 1372918.6) | 0 (0, 3463413448.91) | 0 (0, 17790848869000755200) | 0 (0, 680506.67) | 0 (0, 508515.3) | 0 (0, 118421.14) |
| XiaoZhiLing |  |  |  | 0 (0, 16241528698388697088) | 19.88 (0, 3.41219999972084e+22) | 0.52 (0.01, 16.17) | 0.4 (0.02, 4.07) | 0.1 (0, 1.27) |
| FanTengZhi |  |  |  |  | 2635510.48 (0, 1.41108547814605e+35) | 3125.12 (0, 5.95823239083266e+26) | 2173.6 (0, 9.09571605722529e+26) | 520.11 (0, 1.72785407803487e+26) |
| Phenol |  |  |  |  |  | 0.02 (0, 510706967109294) | 0.02 (0, 287281686483602) | 0 (0, 63996629443158.5) |
| Traditional hemorrhoidectomy |  |  |  |  |  |  | 0.74 (0.03, 13.45) | 0.18 (0.01, 2.62) |
| PPH |  |  |  |  |  |  |  | 0.25 (0.01, 2.54) |

CI: confidence interval; PPH, procedure for prolapse and hemorrhoids; RBL, rubber band ligation.

**Table S8. Odds ratio and 95% CI of the complication rate (urinary retention) for each treatment on the first row (red color) as compared with each treatment on the first column (green color)**

|  | Polidocanol foam | XiaoZhiLing | Phenol | Coagulation | Traditional hemorrhoidectomy | PPH | RBL |
| --- | --- | --- | --- | --- | --- | --- | --- |
| Polidocanol liquid | 0.15 (0, 5.04) | 5.9 (0.25, 178.04) | 2.37 (0.07, 81.19) | 0 (0, 453066034141053120) | 7.63 (0.97, 98.67) | 2.66 (0.12, 73.98) | 3.56 (0.64, 26.11) |
| Polidocanol foam |  | 40.52 (0.43, 13947.04) | 16.1 (0.12, 5394.33) | 0 (0, 3138274853136107008) | 53.91 (1.01, 12275.81) | 18.78 (0.18, 5599.95) | 24.25 (0.63, 3821.2) |
| XiaoZhiLing |  |  | 0.4 (0, 28.43) | 0 (0, 67223570315107032) | 1.32 (0.06, 33.84) | 0.46 (0.01, 16.92) | 0.62 (0.02, 14.67) |
| Phenol |  |  |  | 0 (0, 165175796562325664) | 3.3 (0.12, 141.35) | 1.15 (0.01, 108.22) | 1.51 (0.06, 46.29) |
| Coagulation |  |  |  |  | 67824125.4 (0, 8.1380220321753e+37) | 23623346.88 (0, 2.4641995693926e+37) | 31742479.15 (0, 3.9338118720721e+37) |
| Traditional hemorrhoidectomy |  |  |  |  |  | 0.35 (0.01, 11.25) | 0.47 (0.03, 4.79) |
| PPH |  |  |  |  |  | PPH | 1.35 (0.05, 39.31) |

CI: confidence interval; PPH, procedure for prolapse and hemorrhoids; RBL, rubber band ligation.

**Table S9. Odds ratio and 95% CI of the complication (rectal stricture) rate for each treatment on the first row (red color) as compared with each treatment on the first column (green color)**

|  | Polidocanol foam | Phenol | Traditional hemorrhoidectomy | PPH | RBL |
| --- | --- | --- | --- | --- | --- |
| Polidocanol liquid | 0 (0, 0.73) | 0 (0, 8.09) | 4.42 (0.21, 234.3) | 0 (0, 132894456813.8) | 0 (0, 2.5) |
| Polidocanol foam |  | 698.5 (0, 938629072774153984) | 100179920.28 (3.75, 1.96796934711774e+21) | 938.08 (0, 3.22913135324175e+22) | 5 (0, 4118663099773803) |
| Phenol |  |  | 19932.42 (1.3, 3363143600713685) | 0.9 (0, 215966948185431360) | 0 (0, 383320099.32) |
| Traditional hemorrhoidectomy |  |  |  | 0 (0, 17620557787.78) | 0 (0, 0.29) |
| PPH |  |  |  |  | 0 (0, 8.56532902497913e+22) |

CI: confidence interval; PPH, procedure for prolapse and hemorrhoids; RBL, rubber band ligation.

**Table S10. Subgroup of internal hemorrhoids: odds ratio and 95% CI of the cure rate for each treatment on the first row (red color) as compared with each treatment on the first column (green color)**

|  | Polidocanol liquid | Polidocanol foam | Dextrose | ShaoBei | XiaoZhiLing | FanTengZhi | Phenol | Coagulation | Traditional hemorrhoidectomy | PPH |
| --- | --- | --- | --- | --- | --- | --- | --- | --- | --- | --- |
| Polidocanol liquid |  | 2.91 (2.07, 4.05) | 0.45 (0.09, 2.08) | 1.13 (0.34, 3.71) | 0.5 (0.34, 0.73) | 0.77 (0.15, 4.02) | 0.45 (0.25, 0.83) | 0.65 (0.27, 1.56) | 0.57 (0.33, 0.99) | 0.6 (0.31, 1.14) |
| Polidocanol foam |  |  | 0.16 (0.03, 0.74) | 0.39 (0.11, 1.33) | 0.17 (0.11, 0.28) | 0.27 (0.05, 1.42) | 0.16 (0.08, 0.3) | 0.22 (0.09, 0.56) | 0.2 (0.11, 0.37) | 0.21 (0.1, 0.43) |
| Dextrose |  |  |  | 2.49 (0.35, 17.95) | 1.11 (0.23, 5.53) | 1.71 (0.18, 16.56) | 1 (0.23, 4.77) | 1.44 (0.29, 7.68) | 1.27 (0.26, 6.44) | 1.33 (0.25, 7.34) |
| ShaoBei |  |  |  |  | 0.45 (0.15, 1.39) | 0.68 (0.1, 4.91) | 0.4 (0.11, 1.54) | 0.58 (0.13, 2.51) | 0.5 (0.14, 1.88) | 0.53 (0.14, 2.07) |
| XiaoZhiLing |  |  |  |  |  | 1.53 (0.31, 7.7) | 0.9 (0.46, 1.81) | 1.3 (0.5, 3.4) | 1.14 (0.63, 2.11) | 1.19 (0.57, 2.47) |
| FanTengZhi |  |  |  |  |  |  | 0.59 (0.1, 3.39) | 0.85 (0.13, 5.43) | 0.74 (0.13, 4.09) | 0.78 (0.13, 4.44) |
| Phenol |  |  |  |  |  |  |  | 1.44 (0.68, 2.92) | 1.26 (0.6, 2.64) | 1.32 (0.55, 3.07) |
| Coagulation |  |  |  |  |  |  |  |  | 0.87 (0.33, 2.43) | 0.92 (0.32, 2.72) |
| Traditional hemorrhoidectomy |  |  |  |  |  |  |  |  |  | 1.05 (0.45, 2.42) |

CI: confidence interval; PPH, procedure for prolapse and hemorrhoids.

**Table S11. Subgroup of grade I/II hemorrhoids: odds ratio and 95% CI of the cure rate for each treatment on the first row (red color) as compared with each treatment on the first column (green color)**

|  | Polidocanol foam | XiaoZhiLing | FanTengZhi | Phenol | Coagulation | Traditional hemorrhoidectomy | RBL |
| --- | --- | --- | --- | --- | --- | --- | --- |
| Polidocanol liquid | 2.71 (1.16, 5.97) | 0.67 (0.24, 1.97) | 1.04 (0.13, 8.2) | 0.35 (0.12, 1.01) | 0.52 (0.14, 1.79) | 0.85 (0.16, 4.82) | 0.93 (0.36, 2.36) |
| Polidocanol foam |  | 0.25 (0.07, 1.03) | 0.38 (0.05, 3.75) | 0.13 (0.04, 0.52) | 0.19 (0.04, 0.84) | 0.31 (0.05, 2.22) | 0.34 (0.1, 1.24) |
| XiaoZhiLing |  |  | 1.52 (0.28, 9) | 0.52 (0.12, 2.3) | 0.77 (0.13, 3.75) | 1.27 (0.17, 9.11) | 1.38 (0.33, 5.62) |
| FanTengZhi |  |  |  | 0.34 (0.03, 3.34) | 0.5 (0.04, 5.14) | 0.82 (0.06, 11.61) | 0.89 (0.09, 7.99) |
| Phenol |  |  |  |  | 1.48 (0.59, 3.19) | 2.42 (0.56, 10.81) | 2.64 (1.18, 5.82) |
| Coagulation |  |  |  |  |  | 1.63 (0.34, 9.54) | 1.78 (0.67, 5.4) |
| Traditional hemorrhoidectomy |  |  |  |  |  |  | 1.09 (0.23, 4.74) |

CI: confidence interval; RBL, rubber band ligation.

**Table S12. Subgroup of grade I/II/III hemorrhoids: odds ratio and 95% CI of the recurrence rate for each treatment on the first row (red color) as compared with each treatment on the first column (green color)**

|  | Polidocanol foam | Dextrose | ShaoBei | XiaoZhiLing | FanTengZhi | Phenol | Coagulation | Traditional hemorrhoidectomy | PPH | RBL |
| --- | --- | --- | --- | --- | --- | --- | --- | --- | --- | --- |
| Polidocanol liquid | 3.03 (1.93, 4.61) | 0.47 (0.09, 2.52) | 1.53 (0.49, 4.83) | 0.51 (0.26, 0.97) | 0.79 (0.12, 5.23) | 0.48 (0.23, 1.01) | 0.65 (0.23, 1.83) | 0.76 (0.36, 1.61) | 0.57 (0.24, 1.35) | 1.07 (0.7, 1.63) |
| Polidocanol foam |  | 0.16 (0.03, 0.87) | 0.51 (0.15, 1.71) | 0.17 (0.08, 0.36) | 0.26 (0.04, 1.83) | 0.16 (0.07, 0.37) | 0.22 (0.07, 0.64) | 0.25 (0.11, 0.6) | 0.19 (0.07, 0.49) | 0.35 (0.21, 0.6) |
| Dextrose |  |  | 3.29 (0.43, 24.12) | 1.08 (0.18, 6.3) | 1.67 (0.14, 20.75) | 1.02 (0.21, 5.11) | 1.37 (0.23, 8.21) | 1.61 (0.27, 9.78) | 1.2 (0.19, 7.92) | 2.26 (0.45, 11.79) |
| ShaoBei |  |  |  | 0.33 (0.13, 0.85) | 0.51 (0.07, 3.8) | 0.32 (0.09, 1.19) | 0.42 (0.09, 1.93) | 0.49 (0.14, 1.73) | 0.37 (0.09, 1.48) | 0.7 (0.21, 2.25) |
| XiaoZhiLing |  |  |  |  | 1.53 (0.28, 8.98) | 0.94 (0.39, 2.41) | 1.27 (0.4, 4.08) | 1.48 (0.66, 3.44) | 1.12 (0.41, 3.07) | 2.09 (1.04, 4.25) |
| FanTengZhi |  |  |  |  |  | 0.62 (0.08, 4.34) | 0.83 (0.1, 6.71) | 0.96 (0.14, 6.48) | 0.72 (0.1, 5.45) | 1.36 (0.2, 9.01) |
| Phenol |  |  |  |  |  |  | 1.35 (0.57, 3.03) | 1.57 (0.61, 4.04) | 1.19 (0.39, 3.55) | 2.2 (1.13, 4.29) |
| Coagulation |  |  |  |  |  |  |  | 1.16 (0.35, 4.05) | 0.88 (0.24, 3.31) | 1.65 (0.62, 4.4) |
| Traditional hemorrhoidectomy |  |  |  |  |  |  |  |  | 0.76 (0.24, 2.26) | 1.41 (0.64, 3.1) |
| PPH |  |  |  |  |  |  |  |  |  | 1.87 (0.76, 4.65) |

CI: confidence interval; PPH, procedure for prolapse and hemorrhoids; RBL, rubber band ligation.

**Figure S1. Convergence diagnostics of the network meta-analysis for the cure rate of hemorrhoids.**

Shrinkage factor plot for the convergence assessment of the Bayesian model. The black line represents the shrinkage factor (potential scale reduction factor) as a function of the number of iterations, while the horizontal line indicates the reference threshold at 1.0. Convergence is considered adequate when the shrinkage factor declines to and stabilizes near 1.0 (or below 1.05) as the number of iterations increases. In this analysis, all treatment comparisons for hemorrhoid cure rate demonstrated satisfactory convergence, indicating that the Markov chains have mixed well and the model estimates are reliable.

**Figure S2.Network meta-analysis of recurrence rate for hemorrhoid treatments**

(A) Network plot. Nodes represent treatments. Edges represent direct comparisons; edge width is proportional to the number of studies. (B) Forest plot shows the odds ratios (OR) with 95% credible intervals for each treatment versus polidocanol liquid. OR > 1 indicates that the treatment has a higher probability of recurrence compared with polidocanol liquid. (C) Ranking probability plot. Darker bars indicate higher ranks, while lighter/gray bars indicate lower ranks. The darkest bar corresponds to rank 1.

**Figure S3.Network meta-analysis of satisfaction rate for hemorrhoid treatments**

(A) Network plot. Nodes represent treatments. Edges represent direct comparisons; edge width is proportional to the number of studies. (B) Forest plot shows the odds ratios (OR) with 95% credible intervals for each treatment versus polidocanol liquid. OR > 1 indicates that the treatment has a higher probability of satisfaction compared with polidocanol liquid. (C) Ranking probability plot. Darker bars indicate higher ranks, while lighter/gray bars indicate lower ranks. The darkest bar corresponds to rank 1.

**Figure S4.Network meta-analysis of overall complication rate for hemorrhoid treatments**

(A) Network plot. Nodes represent treatments. Edges represent direct comparisons; edge width is proportional to the number of studies. (B) Forest plot shows the odds ratios (OR) with 95% credible intervals for each treatment versus polidocanol liquid. OR > 1 indicates that the treatment has a higher probability of complication compared with polidocanol liquid. (C) Ranking probability plot. Darker bars indicate higher ranks, while lighter/gray bars indicate lower ranks. The darkest bar corresponds to rank 1.

**Figure S5.Network meta-analysis of the complication rate (pain) for hemorrhoid treatments**

(A) Network plot. Nodes represent treatments. Edges represent direct comparisons; edge width is proportional to the number of studies. (B) Forest plot shows the odds ratios (OR) with 95% credible intervals for each treatment versus polidocanol liquid. OR > 1 indicates that the treatment has a higher probability of pain compared with polidocanol liquid. (C) Ranking probability plot. Darker bars indicate higher ranks, while lighter/gray bars indicate lower ranks. The darkest bar corresponds to rank 1.

**Figure S6.Network meta-analysis of the complication rate (bleeding) for hemorrhoid treatments**

(A) Network plot. Nodes represent treatments. Edges represent direct comparisons; edge width is proportional to the number of studies. (B) Forest plot shows the odds ratios (OR) with 95% credible intervals for each treatment versus polidocanol liquid. OR > 1 indicates that the treatment has a higher probability of bleeding compared with polidocanol liquid. (C) Ranking probability plot. Darker bars indicate higher ranks, while lighter/gray bars indicate lower ranks. The darkest bar corresponds to rank 1.

**Figure S7. Network meta-analysis of the complication rate (fever, infection, or abscess) for hemorrhoid treatments**

(A) Network plot. Nodes represent treatments. Edges represent direct comparisons; edge width is proportional to the number of studies. (B) Forest plot shows the odds ratios (OR) with 95% credible intervals for each treatment versus polidocanol liquid. OR > 1 indicates that the treatment has a higher probability of fever, infection, or abscess compared with polidocanol liquid. (C) Ranking probability plot. Darker bars indicate higher ranks, while lighter/gray bars indicate lower ranks. The darkest bar corresponds to rank 1.

**Figure S8. Network meta-analysis of the complication rate (urinary retention) for hemorrhoid treatments**

(A) Network plot. Nodes represent treatments. Edges represent direct comparisons; edge width is proportional to the number of studies. (B) Forest plot shows the odds ratios (OR) with 95% credible intervals for each treatment versus polidocanol liquid. OR > 1 indicates that the treatment has a higher probability of urinary retention compared with polidocanol liquid. (C) Ranking probability plot. Darker bars indicate higher ranks, while lighter/gray bars indicate lower ranks. The darkest bar corresponds to rank 1.

**Figure S9. Network meta-analysis of the complication rate (rectal stricture) for hemorrhoid treatments**

(A) Network plot. Nodes represent treatments. Edges represent direct comparisons; edge width is proportional to the number of studies. (B) Forest plot shows the odds ratios (OR) with 95% credible intervals for each treatment versus polidocanol liquid. OR > 1 indicates that the treatment has a higher probability of rectal stricture compared with polidocanol liquid. (C) Ranking probability plot. Darker bars indicate higher ranks, while lighter/gray bars indicate lower ranks. The darkest bar corresponds to rank 1.

**Figure S10. Subgroup of internal hemorrhoids: network meta-analysis of the cure rate for hemorrhoid treatments**

(A) Network plot. Nodes represent treatments. Edges represent direct comparisons; edge width is proportional to the number of studies. (B) Forest plot shows the odds ratios (OR) with 95% credible intervals for each treatment versus polidocanol liquid. OR > 1 indicates that the treatment has a higher probability of cure compared with polidocanol liquid. (C) Ranking probability plot. Darker bars indicate higher ranks, while lighter/gray bars indicate lower ranks. The darkest bar corresponds to rank 1.

**Figure S11. Subgroup of grade I/II hemorrhoids: network meta-analysis of the cure rate for hemorrhoid treatments**

(A) Network plot. Nodes represent treatments. Edges represent direct comparisons; edge width is proportional to the number of studies. (B) Forest plot shows the odds ratios (OR) with 95% credible intervals for each treatment versus polidocanol liquid. OR > 1 indicates that the treatment has a higher probability of cure compared with polidocanol liquid. (C) Ranking probability plot. Darker bars indicate higher ranks, while lighter/gray bars indicate lower ranks. The darkest bar corresponds to rank 1.

**Figure S12. Subgroup of grade I/II/III hemorrhoids: network meta-analysis of the cure rate for hemorrhoid treatments**

(A) Network plot. Nodes represent treatments. Edges represent direct comparisons; edge width is proportional to the number of studies. (B) Forest plot shows the odds ratios (OR) with 95% credible intervals for each treatment versus polidocanol liquid. OR > 1 indicates that the treatment has a higher probability of cure compared with polidocanol liquid. (C) Ranking probability plot. Darker bars indicate higher ranks, while lighter/gray bars indicate lower ranks. The darkest bar corresponds to rank 1.

**References**

1.	Varma JS, Chung SC, Li AK. Prospective randomised comparison of current coagulation and injection sclerotherapy for the outpatient treatment of haemorrhoids. International journal of colorectal disease. 1991;6(1):42-5.

2.	Walker AJ, Leicester RJ, Nicholls RJ, Mann CV. A prospective study of infrared coagulation, injection and rubber band ligation in the treatment of haemorrhoids. International journal of colorectal disease. 1990;5(2):113-6.

3.	Ambrose NS, Morris D, Alexander-Williams J, Keighley MR. A randomized trial of photocoagulation or injection sclerotherapy for the treatment of first- and second-degree hemorrhoids. Diseases of the colon and rectum. 1985;28(4):238-40.

4.	Gartell PC, Sheridan RJ, McGinn FP. Out-patient treatment of haemorrhoids: a randomized clinical trial to compare rubber band ligation with phenol injection. The British journal of surgery. 1985;72(6):478-9.

5.	Cheng FC, Shum DW, Ong GB. The treatment of second degree haemorrhoids by injection, rubber band ligation, maximal anal dilatation, and haemorrhoidectomy: a prospective clinical trial. The Australian and New Zealand journal of surgery. 1981;51(5):458-62.

6.	Greca F, Hares MM, Nevah E, Alexander-Williams J, Keighley MR. A randomized trial to compare rubber band ligation with phenol injection for treatment of haemorrhoids. The British journal of surgery. 1981;68(4):250-2.

7.	Kanellos I, Goulimaris I, Christoforidis E, Kelpis T, Betsis D. A comparison of the simultaneous application of sclerotherapy and rubber band ligation, with sclerotherapy and rubber band ligation applied separately, for the treatment of haemorrhoids: a prospective randomized trial. Colorectal disease : the official journal of the Association of Coloproctology of Great Britain and Ireland. 2003;5(2):133-8.

8.	Moser KH, Mosch C, Walgenbach M, Bussen DG, Kirsch J, Joos AK, et al. Efficacy and safety of sclerotherapy with polidocanol foam in comparison with fluid sclerosant in the treatment of first-grade haemorrhoidal disease: a randomised, controlled, single-blind, multicentre trial. International journal of colorectal disease. 2013;28(10):1439-47.

9.	Akindiose C, Alatise OI, Arowolo OA, Agbakwuru AE. Evaluation of two injection sclerosants in the treatment of symptomatic haemorrhoids in Nigerians. The Nigerian postgraduate medical journal. 2016;23(3):110-5.

10.	Abiodun AA, Alatise OI, Okereke CE, Adesunkanmi AK, Eletta EA, Gomna A. Comparative study of endoscopic band ligation versus injection sclerotherapy with 50% dextrose in water, in symptomatic internal haemorrhoids. The Nigerian postgraduate medical journal. 2020;27(1):13-20.

11.	Mishra S, Sahoo AK, Elamurugan TP, Jagdish S. Polidocanol versus phenol in oil injection sclerotherapy in treatment of internal hemorrhoids: A randomized controlled trial. The Turkish journal of gastroenterology : the official journal of Turkish Society of Gastroenterology. 2020;31(5):378-83.

12.	Salgueiro P, Garrido M, Santos RG, Pedroto I, Castro-Poças FM. Polidocanol Foam Sclerotherapy Versus Rubber Band Ligation in Hemorrhoidal Disease Grades I/II/III: Randomized Trial. Diseases of the colon and rectum. 2022;65(7):e718-e27.

13.	Cestaro G, De Rosa M, Mosella F, Amato B, Gentile M. Rubber band ligation versus endoscopic injection sclerotherapy for symptomatic second-degree hemorrhoids: a prospective randomised trial. G Chir 2013;34(11):341-3.

14.	Shen F, Qu CY, Zhang Y, et al. Therapeutic effects of endoscopic foam sclerotherapy for bleeding internal hemorrhoids. Chin J Dig Endosc 2019;36(12):917-22.

15.	Xin Y. Clinical effect and safety feasibility of lauromacrogol injection under colonoscopy with a transparent cap in the treatment of hemorrhoidal internal bleeding. J Prim Med Forum 2025;29(23):70-72.

16.	Cai XY, Shen Y, Gong MJ, et al. Comparison of efficacy and postoperative complications of lauromacrogol foam sclerosant and lauromacrogol injection in patients with internal hemorrhoids. J Kunming Med Univ 2022;43(12):137-41.

17.	Zhang JE, Zhao PX, Liu JL, et al. Clinical effect observation of FanTengZhi and two other different drugs injection in the treatment of mixed hemorrhoids. Home Med 2018;110(8):73.

18.	Kong XQ, Gao JH, Sun SZ, et al. Clinical effect of Fanteng sclerosing agent for hemorrhoids in the treatment of grade I to II internal hemorrhoids. J Colorect Anal Surg 2022;28(6):588-92.

19.	Liang QS, Liu CW. Clinical observation of lauromacrogol through anoscopic injection in the treatment of hemorrhagic internal hemorrhoids. China Prac Med 2015;10(11): 26-27.

20.	Lian SX, Zhang Y, Zou ZY, et al. Comparison on the effects of internal hemorrhoids treated with rubber band ligation and with sclerosing agent injection. Chin J Coloproctol 2016;36(3):21-23.

21.	Li F, Tan LX, Zhao YH, et al. Sclerotherapy with lauromacrogol injection under colonoscope in the treatment of internal hemorrhoids. J China Prescription Drug 2020;18(6):86-87.

22.	Liu H, Ma J, Kang D, et al. Clinical application of lauromacrogol injection under colonoscope in the treatment of internal hemorrhoids. Women's Health 2024;24:54-56.

23.	Lin H, Li HZ, Li Q, et al. Effect of lauryl alcohol sclerotherapy on clinical efficacy, cellular immune status and adverse reactions in patients with internal hemorrhoids under colonoscopy. Chin Foreign Med Res 2021;19(13):23-6.

24.	Huo ZN, Ma SY. Clinical comparison of Xiaozhiling hemorrhoids injection under colonoscopy and traditional external stripping and internal ligation in the treatment of grade II and III mixed hemorrhoids. World J Complex Med 2022;8(12):77-80.

25.	Tan TT, Liao W. Clinical observation of lauromacrogol through colonoscopic injection in the treatment of internal hemorrhoids. Diet Health 2018;5(8):56-7.

26.	Liu L, Gu JH. Clinical effect research of sclerotherapy injection of polydocanol foam in treatment of internal hemorrhoids at different stages. Smart Healthcare 2024;24(24):40-2.

27.	Li S, Ju L, Lin XW. Clinical observation of polidocanol injection in the treatment of internal hemorrhoids. J North Pharm 2025;22(7):69-71.

28.	Ai W, Nurmamat Y, Abdurayim A, et al. Clinical effect analysis of polidocanol foam sclerosing agent versus polidocanol liquid sclerosing agent in the treatment of internal hemorrhoidal bleeding. Fertil Health 2020;15:149.

29.	Tang WH, Lei JC, Yang QF. Comparison of efficacy between polidocanol foam sclerotherapy and liquid sclerotherapy in the treatment of bleeding internal hemorrhoids. Zhejiang Practical Medicine 2021;26(1):66-68.

30.	Ai W, Nurmamat Y, Abdurayim A, et al. Clinical effect analysis of lauromacrogol foam sclerotherapy in the treatment of hemorrhoidal disease. Yiyao Qianyan 2021;11(20):28-30.

31.	Lei QJ, Zhang YQ, He XD. Clinical observation of lauromacrogol sclerotherapy in the treatment of hemorrhagic internal hemorrhoids. Modern Journal of Integrated Traditional Chinese and Western Medicine 2017;26(10):1116-7.

32.	Liu H. Observation on the efficacy of lauromacrogol sclerotherapy in the treatment of internal hemorrhoidal bleeding. Scientific & Technical Information of Gansu 2022;51(11):86-92.

33.	Wang F, Gong XC. Efficacy and Safety of Lauromacrogol Sclerotherapy for Internal Hemorrhoids Bleeding. Chinese Journal of Coloproctology 2018;38(2):30-32.

34.	Xu Q. Clinical Efficacy of Lauromacrogol Sclerotherapy for Internal Hemorrhoids. Journal of Coloproctology 2014;34(10):17-8.

35.	Zhang YK. Internal hemorrhoids treated with lauromacrogol injection: effect observation on 45 cases. Chin J Coloproctol 2019;39(1):11-2.

36.	Dong J. Comparison of clinical effect between endoscopic rubber band ligation and sclerotherapy injection in treatment of hemorrhagic internal hemorrhoids. Journal of Chinese and Foreign Medicine and Pharmacy Research 2025;4(14):10-12.

37.	Chen CW, Chen ZT, Jiang ZB, et al. Effect of endoscopic local injection of lauromacrogol in the treatment of stage Ⅱ to Ⅲ hemorrhoids. World Journal of Complex Medicine 2023;9(9):176-88.

38.	Tang SJ, Xia LQ, Xiang F, et al. Clinical effect of endoscopic lauromacrogol foam sclerotherapy in the treatment of internal hemorrhoids. Advice for Health 2021;(7):58.

39.	Xiao M, Yu XH, Xu C, et al. Comparison of endoscopic sclerotherapy with polycinnamol solution and foam injection in the treatment of grade II hemorrhagic internal hemorrhoids. Journal of Chinese Physician 2023;25(6):819-23.

40.	Zhang YJ, Zhang FY, Shen F, et al. Efficacy of endoscopic foam sclerosant versus liquid sclerotherapy for degree I-III internal hemorrhoids: a randomized controlled trial. China Journal of Endoscopy 2023;29(7):48-55.

41.	Wei JQ. The Effects of Endoscopic Ligation, Endoscopic Sclerotherapy Injection, and Stapler Hemorrhoidectomy on Perioperative Indicators, Pain, and Complications in Elderly Patients with Grade II to III Internal Hemorrhoids. China Medical Device Information 2023;29(21):122-124.

42.	Ye Y, Liang YF, Sun XZ, et al. A comparative study of endoscopic ligation, cap-assisted endoscopic injection of polidocanol foam or stock solution sclerotherapy in the treatment of internal hemorrhoids. Modern Digestion & Intervention 2022;27(2):185-9.

43.	Lu SS, Hu MQ, Tao MG. Efficacy of endoscopic ligation and polidocanol sclerotherapy in the treatment of stage II and III internal hemorrhoids. Zhejiang Clinical Medical Journal 2024;26(10):1519-1523.

44.	Chen W, Fan W, Wu W. Comparison of the efficacy of endoscopic ligation and sclerosing injection of Lauromacrogol in the treatment of stage II and III hemorrhoids. China Modern Medicine 2022;29(34):140-146.

45.	Che ZY. Comparison of therapeutic efficacy between endoscopic rubber band ligation and cap-assisted endoscopic sclerotherapy for grade Ⅱ internal hemorrhoids. Jian Kang Zhong Gao 2024;22(22):13-16.

46.	Zhong T, Pang WJ, Wang L, et al. Clinical analysis of 53 cases of internal hemorrhoids treated with minimally invasive endoscopy. International Journal of Digestive Diseases 2022;42(6):378-82.

47.	Yu TJ, Jiang L, Li WS, et al. Clinical efficacy of Xiaozhiling injection in treatment of 60 cases of internal hemorrhoids under endoscope. Chinese Journal of Coloproctology 2023;43(12):23-25.

48.	Lyu MG, Pan XZ. Clinical effect of endoscopic sclerotherapy for internal hemorrhoids. Chinese and Foreign Medical Research 2021;19(5):29-31.

49.	Yuan MH, She CH, Zhu XL. Comparison of the Efficacy and Recurrence Risk of Internal Hemorrhoids Treated by Endoscopic Sclerotherapy Injection and Ligation. Systems Medicine 2023;8(22):96-99.

50.	Ren MF, Zhang HL, Wang W, et al. Observation on the efficacy and safety of endoscopic sclerotherapy versus procedure for prolapse and hemorrhoids in the treatment of internal hemorrhoids. Guizhou Medical Journal 2023;47(10):1615-6.

51.	Chen JY, Lei HQ, Han YF. Comparison of efficacy between foam sclerotherapy and lauromacrogol original liquid sclerotherapy in transparent cap-assisted endoscopic treatment of internal hemorrhoids. Heilongjiang Journal of Traditional Chinese Medicine 2021; (3): 437-8.

52.	Feng YN, Wang AB, Tong L, et al. Clinical Study of Shaobei Injection with Xiaozhiling in the Treatment of Mixed Hemorrhoids. Journal of Basic Chinese Medicine 2016;22(10):1369-71.

53.	Wang JP, Yang XD, Xu AG, et al. Clinical observation of Shaobei injection in the treatment of two, three internal hemorrhoids: on 180 cases report. Chin J of Clinical Rational Drug Use 2013;11(2A):23-24.

54.	Wang YF. Observation on the therapeutic effects of ShaoBei injection for internal hemorrhoids and varicose mixed hemorrhoids. Chinese Journal of Coloproctology 2008;28(4):33-4.

55.	Liu DB, Tang ZJ. Clinical observation on 32 cases of hemorrhoids treated by Shaobei Injection. Hunan Journal of Traditional Chinese Medicine 2008;24(3):27-8.

56.	Liao M, Deng HC, Chen XY, et al. Clinical study on the treatment of 385 cases of hemorrhoids with astringent and stasis-removing method. Chinese Journal for Clinicians 2008;36(4):54.

57.	Chen HD, Luo ZM, Pan JR. Safety and efficacy of sclerotherapy with lauromacrogol under transparent cap-assisted colonoscopy in the treatment of bleeding internal hemorrhoids. Guizhou Medical Journal 2023;47(5):734-5.

58.	Gui G, Yi J, Lu ZY, et al. Clinical efficacy observation of cap-assisted endoscopic modified foam sclerotherapy and ligation in the treatment of internal hemorrhoids. Medical Equipment 2024;37(18):70-72.

59.	Huang HC, Zhang HB, Meng M, et al. Transparent cap assisted endoscopic foam sclerotherapy for internal hemorrhoids: a preliminary study. Chinese Journal of Colorectal Diseases. 2020;9(6):621-4.

60.	Yan F, Chao S, Li Y, et al. Clinical effect of transparent cap-assisted endoscopic foam sclerotherapy in the treatment of patients with internal hemorrhoids. J Mod Med Health 2025;41(10):2358-61.

61.	Xiong Y, Xie CF, Yang Y, et al. Logistic regression analysis of the difference in curative effect between transparent cap - assisted endoscopic sclerotherapy and traditional surgery for hemorrhoids and poor prognosis of patients. Journal of Clinical and Experimental Medicine 2022;21(7):755-758.

62.	Zheng L, Huang YQ, Yao L, et al. Evaluation of the efficacy of transparent cap assisted endoscopic sclerotherapy in the treatment of internal hemorrhoids. Laboratory Medicine and Clinic 2024;21(5):613-6.

63.	Zhou HR, Li Y. Safety analysis of transparent cap assisted endoscopic sclerotherapy for hemorrhoids. China Continuing Medical Education 2021;13(35):137-41.

64.	Zhou W, Ma X, Li JH. Efficacy and Safety of Cap-assisted Endoscopic Lauromacrogol Sclerotherapy for Grade I - III Internal Hemorrhoids. Journal of Hubei University of Science and Technology (Medical Sciences) 2021;35(1):25-28.

65.	Zhao R, Tan XM, Chen H, et al. Curative effect analysis of lauromacrogol foam sclerotherapy for internal hemorrhoids under painless colonoscopy. Military Medical Journal of Southeast China 2022;24(6):578-81.

66.	Ni HH, Lei HQ, Han YF. Application of lauromacrogol injection in the treatment of internal hemorrhoids under painless colonoscopy. Heilongjiang Journal of Traditional Chinese Medicine 2020;2020(5):45.
